# The impact of modern agricultural technology promotion and adoption on agri-food system resilience

**DOI:** 10.3389/fnut.2025.1677104

**Published:** 2025-10-02

**Authors:** Yuting Zhang, Juan Shang, Ziling Yu

**Affiliations:** ^1^School of Economics and Management, Xidian University, Xi’an, China; ^2^School of Economics and Management, Xinjiang University, Urumqi, China

**Keywords:** modern agricultural technology, promotion and adoption, National Modern Agricultural Demonstration Zone, Agri-food system resilience, food security

## Abstract

Enhancing agri-food system resilience holds profound strategic significance for ensuring food security in developing countries. This study constructs an evolutionary game model at the county level to theoretically explore the positive effects of modern agricultural technology promotion and adoption on agri-food system resilience. Based on panel data from 2,312 counties in China during the period 2006–2023, and using the National Modern Agricultural Demonstration Zone policy as a quasi-natural experiment, the study employs a multi-period difference-in-differences approach to rigorously investigate the effects and underlying mechanisms of modern agricultural technology promotion and adoption on agri-food system resilience. The empirical findings indicate that: (1) The diffusion of modern agricultural technologies significantly enhances agri-food system resilience, and this conclusion remains robust after a series of consistency checks. (2) Mechanism analysis shows that modern agricultural technology diffusion improves resilience by enhancing agricultural technological innovation capacity, increasing local government attention to agriculture, promoting agricultural financial development, and improving urban–rural coordination. (3) Heterogeneity analysis reveals that the positive impact of modern agricultural technology diffusion is more pronounced in counties with labor outflow, in non-major grain-producing regions, in the second and third batches of National Modern Agricultural Demonstration Zone, in Han Chinese districts, and in counties with higher levels of education. (4) Kernel density analysis demonstrates that modern agricultural technology diffusion has contributed to narrowing the disparities in agri-food system resilience across counties. These findings provide valuable empirical evidence and policy insights for developing countries seeking to bolster agri-food system resilience and safeguard food security.

## Graphical abstract

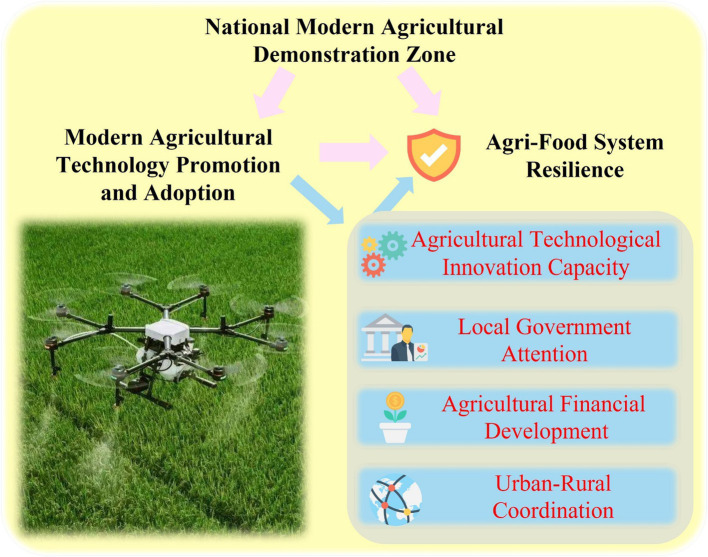

## 1 Introduction

In recent years, the increasing frequency of extreme weather events and the rapidly evolving domestic and international trade environments have posed significant challenges to food security (1). In 2018, for instance, extreme heat and drought conditions caused cereal production in Europe to fall below the average level of the preceding 5 years. Current long-term projections indicate that global temperatures are rising by about 0.2 °C per decade, implying that such declines in crop yields are likely to persist (2). Moreover, rural infrastructure, agricultural machinery, and farm property are also exposed to damage during use, particularly under severe shocks. In such cases, the safety of agricultural production and the stability of agricultural supply chains may suffer devastating impacts (3–5).

Despite the rapid development of many developing countries, the most formidable and pressing challenges remain concentrated in rural areas (6). When major shocks occur, these risks can have devastating impacts on the safety of agricultural production. In response to these persistent external shocks, many developing countries have accelerated the pace of agricultural modernization in an effort to address the growing difficulties and uncertainties in agricultural development. However, agri-food production systems continue to show vulnerability when confronted with such risks (7, 8). Enhancing agri-food system resilience is therefore essential for mitigating disruptions in food supply, maintaining food quality, and ensuring food security (9, 10). Strengthening the capacity of agricultural production to cope with and recover from risks has become an urgent priority. This objective is closely aligned with the concept of agri-food system resilience, which emphasizes the ability of production systems to re-sist disaster-related shocks, adapt quickly, and return to a stable state. Such resilience plays a key role in minimizing the negative effects of uncertainty and risk on food security (11).

Ensuring food security and strengthening agri-food system resilience are essential pathways for promoting comprehensive and sustainable economic development in these regions. Rural modernization is both a fundamental requirement and a necessary condition for building strong agricultural sectors. Achieving this goal requires integrated progress in both agricultural and rural modernization. However, agricultural production is characterized by long cycles and high dependence on natural conditions and climate stability. In an increasingly volatile external environment, enhancing the resilience of agri-food systems has become not only a critical issue but also a complex challenge (2). With the rapid advancement of modern agriculture and the frequent occurrence of “black swan” events, strengthening agri-food system resilience to ensure food security has emerged as a focal point of attention for both policymakers and scholars.

The term “resilience” originates from the Latin word *resilire*, referring to the ability of a system or individual to recover or rebound after experiencing shocks or disturbances (12, 13). While initially used in physics, the concept was later introduced into ecology and, in recent years, has been increasingly applied in the field of agricultural development (3, 14–18). Agri-food systems, as a type of socio-ecological system, embody this concept. A resilient agri-food system can absorb greater levels of disruption while maintaining adaptability and long-term sustainability (19, 20). Agri-food system resilience refers to the capacity of such systems to withstand, recover, adapt, and transform when confronted with disturbances. In the field of resilience research, agri-food systems exhibit distinct systemic characteristics. The large population scale, combined with the coexistence of household-based farming and commercial agricultural production, poses additional difficulties and challenges for strengthening agri-food system resilience (21, 22).

Currently, a considerable number of scholars empirically analyze agri-food system resilience by constructing evaluation indicator frameworks (23). The indicator system approach has become mainstream, with measurement dimensions becoming increasingly well-defined. Existing literature predominantly measures and studies agri-food system resilience from three dimensions: resistance, recovery, and innovation capacity. Regarding the factors influencing the agri-food system resilience, the roles of the digital economy (24), infrastructure development (25, 26), industrial integration (27), collaborative capacity (28), labor force (29), agricultural practices (30), organic farming (31), climate change (32), and policy adjustments (33) have been widely recognized. Existing research consistently demonstrates that these factors play a positive role in enhancing the resilience of the agricultural economy.

In addition, the influence of the number of women farmers and of regions with concentrated ethnic minority populations on agricultural economic resilience should not be overlooked. On the one hand, with the progress of urbanization and industrialization, a large share of rural male labor has migrated to cities, leaving women farmers as the primary agents of agricultural production and livelihood. Their role has become crucial for agricultural development and for strengthening the resilience of agri-food systems. The contribution of women who remain in rural areas has attracted extensive scholarly attention (34–36). Most scholars, using surveys and interviews, have conducted in-depth investigations into how women farmers across different districts affect agricultural resilience (37, 38). Through knowledge sharing, institutional improvements, and access to resources, they have constructed agricultural intervention systems that reduce production risks at the household level and enhance the regional capacity to respond to agricultural shocks (39). When modern agricultural technologies are promoted and adopted, women farmers are able to compensate for physical disadvantages, alleviating the problem of insufficient labor capacity they often face. This effectively prevents the decline in yields that could result from male labor loss, thereby stabilizing agricultural production and further improving agricultural resilience.

On the other hand, districts inhabited predominantly by ethnic minorities exert multiple influences on agricultural resilience through their distinctive cultural traditions, social structures, and geographical environments. In China, such districts are often located in peripheral mountainous areas, plateaus, or ecologically fragile zones, where agricultural systems are characterized by ecological sensitivity, cultural uniqueness, and socioeconomic underdevelopment. In earlier studies, some scholars focused on issues of ethnic hegemony. In recent years, increasing scholarly attention has been devoted to the positive contributions of ethnic minority districts to agricultural development. Yang et al. (40) argued that optimizing the integration of agriculture and tourism can facilitate the complete eradication of poverty and promote sustainable development in these areas. Our research focuses more specifically on the promotion and adoption of modern agricultural technologies and their implications for agri-food system resilience. The diffusion of modern agricultural technologies is expected to enhance the adaptive and transformative capacities of agricultural systems in ethnic minority districts. Such diffusion can alter traditional crop varieties and farming practices that have long been shaped by natural selection, thereby contributing to greater resilience in agri-food systems. Nevertheless, compared with Han-majority districts, ethnic minority districts face greater challenges, require longer time horizons, and exhibit certain delays in improving agri-food system resilience through the adoption of modern agricultural technologies.

Many studies have also recognized the important role of agricultural modernization in enhancing agri-food system resilience (41, 42). Strengthening the promotion and adoption of modern agricultural technologies helps to extend the agricultural innovation chain and accelerate the transformation of scientific and technological achievements (43, 44). It is a key measure for improving agricultural technology and advancing agricultural and rural modernization (45). As the foundation for ensuring the stable supply of food and major agricultural products, agricultural modernization plays a critical role in improving agri-food system resilience. However, farmers often show strong self-selection in adopting agricultural technologies, making it challenging to scientifically evaluate the impact of modern agricultural technology promotion and adoption on agri-food system resilience. Existing literature mainly focuses on the effects of modern agricultural technologies on farmers’ income or the process of technological innovation (46–48). Some studies emphasize how to increase farmers’ adoption rates of modern technologies (49). However, they have overlooked the impact of promoting and applying modern agricultural technologies on agri-food system resilience.

Under increasingly complex external risks, the promotion and adoption of modern agricultural technologies offer new possibilities for effectively addressing uncertainties related to the economy, climate, and environment. In the process of enhancing agri-food system resilience, advancing rural revitalization, and accelerating the development of a strong agricultural sector, modern agricultural technology plays a vital role. Whether the promotion and adoption of modern agricultural technologies can improve agri-food system resilience to mitigate potential losses from external shocks, and what mechanisms are involved in this process, are key questions. Therefore, exploring the relationship between the promotion and adoption of modern agricultural technologies and agri-food system resilience holds significant theoretical and practical value for ensuring food security, effectively responding to risk shocks, and achieving stable production and increased income.

In 2010, the Chinese agricultural sector initiated the pilot program of establishing National Modern Agricultural Demonstration Zone (NMADZ) in batches. This pilot policy primarily focused on the diffusion and application of agricultural technologies, particularly modern agricultural machinery. Since the NMADZ were introduced in stages and gradually expanded to additional regions, the process not only created comparable treatment and control groups but also allowed for the gradual transformation of control groups into treatment groups. Under otherwise comparable conditions, this provided a valuable quasi-natural experiment for scientifically assessing the income-enhancing effects of modern agricultural technology diffusion. However, existing studies on agri-food system resilience in China have largely concentrated on major grain-producing regions, without sufficient attention to county-level heterogeneity or the role of specific influencing factors. Building on the quasi-natural experiment of China’s NMADZ, this study matches pilot program data with county-level statistical data and employs a difference-in-differences (DID) model to rigorously evaluate the effects and mechanisms of modern agricultural technology diffusion on the agri-food system resilience in China. The findings provide valuable empirical evidence and policy insights for strengthening agri-food system resilience and safeguarding food security in developing countries.

The marginal contributions of this paper are mainly reflected in the following three aspects: First, this study presents an innovative research perspective by integrating the promotion and adoption of modern agricultural technologies into the analytical framework of agri-food system resilience. At the theoretical level, it develops a strategic game model among counties to explore interregional interactions. Empirically, using the NMADZ pilot policy as a policy shock, the study employs a multi-period DID model to rigorously assess the impact of modern agricultural technology promotion and adoption on agri-food system resilience. The findings provide novel empirical evidence for effectively enhancing agri-food system resilience under the new development paradigm.

Second, this study investigates the intrinsic mechanisms through which the promotion and adoption of modern agricultural technologies influence agri-food system resilience. It finds that the NMADZ pilot policy significantly enhances resilience by strengthening agricultural innovation capacity, increasing local governments’ focus on agriculture, promoting agricultural financial development, and optimizing urban–rural coordination.

Third, this study goes beyond the conventional approach of measuring agricultural economic resilience solely from the perspectives of resistance, recovery, and innovation. Grounded in the national strategy of accelerating the development of a strong agricultural sector and guided by the need to enhance the agri-food system resilience, it enriches the literature on the impact of modern agricultural technology diffusion on agri-food system resilience. This study provides valuable policy insights for the design of pilot programs for NMADZ in developing countries, accelerating the promotion and adoption of modern agricultural technologies, enhancing agri-food system resilience, and safeguarding food security. It holds significant practical implications for expediting the improvement of agri-food system resilience in developing countries.

## 2 Theoretical analysis and research hypotheses

### 2.1 Direct impact of modern agricultural technology promotion and adoption on agri-food system resilience: an evolutionary game analysis

In the initial stage of establishing the NMADZ, significant heterogeneity existed across counties in their understanding of the program’s objectives and the promotion and adoption of modern agricultural technologies. This variation was further compounded by the lack of comprehensive policy and technical support, resulting in uneven implementation and the absence of a unified standard for evaluating the actual effectiveness of the NMADZ. Moreover, the promotion and adoption of modern agricultural technologies often involve conflicting interests and responsibilities among multiple stakeholders, particularly the tension between pursuing short-term individual gains and enhancing long-term risk resilience. Therefore, the trade-off between benefits and costs becomes a critical factor in the technology promotion and adoption process.

Evolutionary game theory offers unique advantages in examining multi-agent decision-making, as it enables the analysis of both dynamic evolutionary processes and strategic agent choices (50). The implementation of the NMADZ policy is itself a dynamic evolutionary process. This study innovatively applies evolutionary game theory to investigate how the promotion and adoption of modern agricultural technologies under this policy influence agri-food system resilience (51). Drawing on the studies by Zhang et al. (52) and Zhang et al. (53), the evolutionary game process of modern agricultural technology adoption among counties can be described as follows:

#### 2.1.1 Participants and strategies

County 1 and County 2 serve as the two strategic players. Each county has two available strategies: to promote and apply modern agricultural technologies, or not to do so. The payoffs differ depending on the chosen strategy, thus forming a game-theoretic relationship between the two counties. Both counties make decisions simultaneously, with no sequential order of moves.

#### 2.1.2 Payoff matrix

The payoff matrix for the game between County 1 and County 2 is presented in Table 1.

**Table 1:** Payoff matrix between County 1 and County 2.

County 1	County 2	
	Promote	Not promote
Promote	R + S_1_-C_1_; R + S_1_-C_1_	R + S_1_ + S_2_-C_1_-C_2_; R-S_2_
Not promote	R-S_2_; R + S_1_ + S_2_-C_1_-C_2_	R; R

##### 2.1.2.1 Parameter definitions

R > 0: The county’s capacity to withstand risks when using traditional agricultural technologies prior to adopting modern technologies.

S_1_ > 0: The increase in returns from enhanced risk resilience after promoting and applying modern agricultural technologies.

C_1_ > 0: The cost associated with promoting and applying modern agricultural technologies.

S_2_ > 0: The loss in returns incurred by counties that have not promoted or applied modern agricultural technologies when facing risks.

C_2_ > 0: The additional cost incurred when fewer counties promote and apply modern agricultural technologies, increasing the marginal cost of adoption.

As shown in the payoff matrix above: when both County 1 and County 2 promote and apply modern agricultural technologies, each county enhances its risk resilience, gaining an additional benefit of S1 on top of its original risk resistance capacity R. However, this also incurs a cost C_1_ associated with the adoption of modern technologies. In this case, both counties receive a net payoff of R + S_1_-C_1_.

If County 1 promotes and applies modern agricultural technologies while County 2 does not, County 2 experiences a loss of benefits amounting to S_2_, which is effectively transferred to County 1. Meanwhile, County 1 bears an additional cost C_2_ due to being the sole adopter. Thus, County 1’s final payoff is R + S_1_ + S_2_-C_1_-C_2_, while County 2’s payoff is reduced to R-S_2_. The reverse situation yields symmetric results.

If neither County 1 nor County 2 promotes and applies modern agricultural technologies, both retain their original benefits, and each receives a payoff of R.

#### 2.1.3 Evolutionary game process

Let p denote the proportion of counties that promote and apply modern agricultural technologies, and (1−p) denote the proportion of counties that do not. The expected payoff for counties that adopt the technology is denoted as $\mu_{1}$, as shown in Equation (1); while the expected payoff for those that do not adopt is denoted as $\mu_{2}$, as shown in Equation (2). The average payoff across the entire population is represented by μ, as shown in Equation (3).


(1) $$\mu_{1}=p(R+S_{1}-C_{1})+(1-p)(R+S_{1}+S_{2}-C_{1}-C_{2})$$

(2) $$\mu_{2}=p(R-S_{2})+(1-p)R$$

(3) $$\begin{array}{l} \mu =p\mu_{1}+(1-p)\mu_{2}\\ =p(p(R+S_{1}-C_{1})+(1-p)(R+S_{1}+S_{2}-C_{1}-C_{2}))\\ +(1-p)(p(R-S_{2})+(1-p)R) \end{array}$$

Based on the payoffs described above, the corresponding replicator dynamic equation can be formulated as shown in Equation (4):


(4) $$\begin{array}{c} F(p)=\frac{\mathrm{dp}}{\mathrm{dt}}=p(\mu_{1}-\mu )=p(1-p)(\mu_{1}-\mu_{2})\\ =p(1-p)(pC_{2}-C_{1}-C_{2}+S_{1}+S_{2}) \end{array}$$

The replicator dynamic equation yields stable equilibrium solutions, which are as follows:


$$p^{*}=0,p^{*}=1,p^{*}=\frac{C_{1}+C_{2}-S_{1}-S_{2}}{C_{2}}$$

Where $0<\frac{C_{1}+C_{2}-S_{1}-S_{2}}{C_{2}}<1$

The above three equilibrium points do not necessarily ensure system stability; only those that satisfy the condition $F\prime (p^{*})<0$ constitute evolutionarily stable strategies. The corresponding results are calculated as follows:


$$F\prime (0)=S_{1}+S_{2}-C_{1}-C_{2}$$

$$F\prime (1)=C_{1}-S_{1}-S_{2}$$

$$F\prime (\frac{C_{1}+C_{2}-S_{1}-S_{2}}{C_{2}})=\frac{\left(S_{1}+S_{2}-C_{1})(C_{1}+C_{2}-S_{1}-S_{2}\right)}{C_{2}}$$

#### 2.1.4 Analysis of evolutionary game results

**Figure 1:**
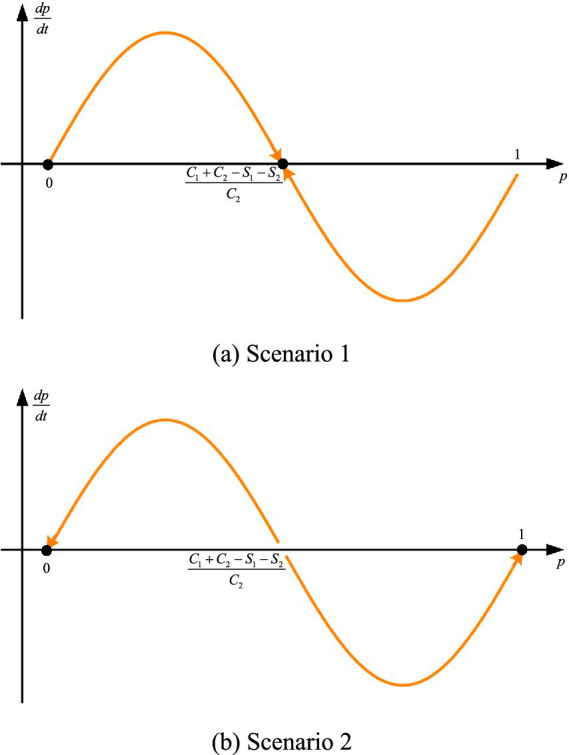
Phase diagrams of the evolutionary game dynamics. **(a)** Scenario 1. **(b)** Scenario 2.

The analysis proceeds based on the underlying assumptions of the game. In the first scenario, when $S_{1}+S_{2}<C_{1}$, with F′(0)<0, F′(1)>0 and $F\prime (\frac{C_{1}+C_{2}-S_{1}-S_{2}}{C_{2}})<0$, the conditions $p^{*}=0$ and $p^{*}=\frac{C_{1}+C_{2}-S_{1}-S_{2}}{C_{2}}$ represent evolutionarily stable strategies in the true sense. This outcome is illustrated in Figure 1a. This indicates that when the cost of promoting and applying modern agricultural technologies is relatively high, counties tend to adopt the more conservative strategy of not promoting such technologies. In the second scenario, when $C_{1}<S_{1}+S_{2}$, with F′(0)<0, F′(1)<0 and $F\prime (\frac{C_{1}+C_{2}-S_{1}-S_{2}}{C_{2}})>0$, the conditions $p^{*}=0$ and $p^{*}=1$ represent evolutionarily stable strategies in the true sense. This outcome is illustrated in Figure 1b. This suggests that when the benefits of promoting and applying modern agricultural technologies are sufficiently high, the mixed strategy becomes unstable, and counties will ultimately converge to a pure strategy. In summary, increasing the risk-resilience benefits associated with the adoption of modern agricultural technologies and reducing the associated costs both contribute to enhancing counties’ ability to withstand risks, thereby strengthening agri-food system resilience at the county level.

In practice, the establishment of NMADZ, has significantly promoted the widespread adoption of modern agricultural technologies by creating the conditions necessary for their routine implementation and by reducing associated adoption costs. Moreover, the integration of production, education, and research has enhanced the capacity of these zones to introduce, integrate, apply, and disseminate new varieties, technologies, and equipment. The promotion and adoption of modern agricultural technologies have accelerated the transformation of scientific and technological achievements, driven agricultural technological progress, optimized industrial structures, and fostered innovations in organizational and management practices. These efforts have substantially improved land productivity, resource-use efficiency, and labor productivity, thereby enhancing the overall quality and efficiency of agricultural development.

Based on the above analysis, this study proposes the following testable hypothesis:

*H1*: *The promotion and adoption of modern agricultural technology contributes to the improvement of Agri-food system resilience.*

### 2.2 The indirect impact of modern agricultural technology promotion and adoption on agri-food system resilience

**Figure 2:**
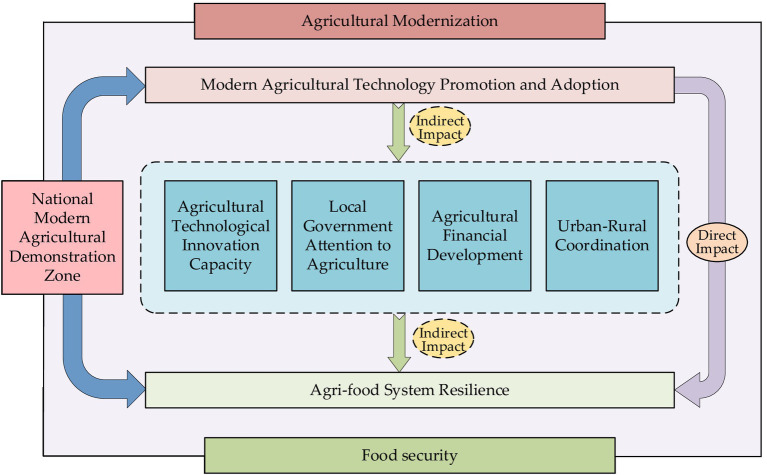
Mechanism of modern agricultural technology promotion and adoption on agri-food system resilience.

Traditional agricultural development has long relied on extensive input of natural resources. However, due to the limitations of these resources, this model has led to increasing resource constraints, weakening the resilience of agriculture. Agricultural modernization represents a shift from traditional labor-intensive practices to modern technology-intensive approaches (54). In the context of modern agricultural technology promotion and adoption, modern technologies are introduced into agricultural production as new types of production inputs (55). According to resource allocation theory, the application of modern agricultural technology helps establish a more efficient resource allocation system, promoting the flow and sharing of key factors such as land and labor (56, 57). This system not only enhances the marginal returns of modern agricultural technologies but also strengthens the interaction among existing agricultural resources. Based on existing literature and the focus of this study, the impact mechanisms of modern agricultural technology promotion and adoption on agri-food system resilience can be divided into four main aspects, as shown in Figure 2.

First, agricultural technology is an essential safeguard for farmers’ production activities. Advances in agricultural technology contribute to improving production efficiency and are crucial for enhancing the productivity, sustainability, and resilience of food production, as well as for strengthening the overall contribution of agriculture to the economy (58). In practice, the Ministry of Agriculture and Rural Affairs of China introduced 80, 100, and 103 recommended technologies for the NMADZ in 2010, 2012, and 2015, respectively. These include soil testing and formula fertilization, straw bio-reactor technology, and ecological crab farming technology. For example, in 2012 the Panshan County NMADZ in Liaoning Province pioneered an efficient three-dimensional ecological integrated farming model that resolved the conflict between fertilization and pesticide application in rice-crab cultivation, achieving a profit of 2,200 yuan per mu and extending demonstration and promotion to an area of more than one million mu both within and outside the province. Therefore, the promotion and adoption of modern agricultural technologies can enhance agricultural technological innovation capacity and thereby strengthen agri-food system resilience. The diffusion of modern agricultural technologies enhances innovation capacity in agriculture, thereby improving the agri-food system resilience. On one hand, according to the theories of technology adoption and innovation diffusion, the advantages of agricultural innovation-such as higher productivity, lower labor costs, and increased yields-promote the free flow of key production factors like labor, capital, and information (59). The application of modern agricultural technology encourages specialization and boosts innovation, generating positive external effects such as better resource allocation, improved industrial structure, and higher production efficiency (60).

On the other hand, diversified industrial development is both a result of agricultural technological innovation and a key strategy for risk dispersion and enhanced adaptability within agricultural systems. Diversification improves the efficiency and effectiveness of recovery after external shocks, strengthening the system’s ability to reorganize resources and restructure to adapt to new environments-that is, increasing the adaptive capacity of agricultural development. Technological innovation also drives changes in farming models, enabling agriculture to quickly shift away from existing growth paths when facing shocks, explore new development directions, and enhance its capacity for innovation and transformation (61). According to endogenous growth theory, agricultural innovation is a fundamental driver of improved agri-food system resilience. Moreover, the NMADZ, supported by favorable policy environments, are well positioned to introduce more advanced production technologies, management practices, and modern agricultural methods. Based on the above analysis, this study proposes the following hypothesis:

*H2a*: *The promotion and adoption of modern agricultural technology enhances Agri-food system resilience by strengthening agricultural innovation capacity.*

Second, the promotion and adoption of modern agricultural technology can enhance agri-food system resilience by increasing local governments’ attention to agriculture. Government decisions are often driven by attention, which reflects policymakers’ focus on specific issues (58, 62). As this attention shifts, government policies tend to adjust accordingly. The direction of attention implies that areas receiving more focus from the government are likely to receive more resources (63, 64). As agricultural modernization and the promotion and adoption of modern agricultural technologies have become critical levers for enhancing agri-food system resilience and ensuring food security, the central government has prioritized agricultural development by highlighting its strategic importance in government work reports and national development plans. The establishment of NMADZ by the central government reflects this strategic orientation. It aligns with broader trends in agricultural development and plays a crucial role in standardizing the development of NMADZ while serving as a model and catalyst for advancing modern agriculture (65). In response, local officials, aiming to align with higher-level priorities and succeed in inter-regional competition, tend to follow the guidance of central government reports and plans. This increases local government attention to agriculture, promotes the implementation of modern agricultural technologies at the local level, and provides strong policy support for improving agri-food system resilience. Based on the above analysis, this study proposes the following hypothesis:

*H2b*: *The promotion and adoption of modern agricultural technology enhances agri-food system resilience by increasing local government attention to agriculture.*

Third, the promotion and adoption of modern agricultural technology can enhance agri-food system resilience by promoting the development of agricultural finance. As agricultural finance continues to develop, its positive impact on agricultural production highlights the importance of inclusive financial development (66). Inclusive finance expands the coverage and accessibility of financial services in agriculture and lowers entry barriers, thereby improving the allocation of financial resources and supporting the growth of key agricultural and major grain-producing regions (67). Agricultural finance can enhance farmers’ capacity to withstand risks by breaking information barriers, alleviating financial constraints, reducing poverty vulnerability, and improving access to financial resources (68–70). From the perspective of information asymmetry, agricultural finance helps reduce search and transaction costs by providing more accessible information on financial products and services. Financial institutions are better able to identify and assess financing needs in rural areas, offering more targeted services to farmers (71).

In addition, risk management theory provides a useful framework for understanding the role of agricultural finance in agricultural production. By reducing uncertainty and easing the financial burden on farmers, agricultural finance supports technological innovation and encourages the adoption of modern agricultural technologies, which in turn enhances agri-food system resilience (72, 73). Moreover, the promotion and adoption of modern agricultural technologies helps develop agricultural finance by encouraging the use of mechanization to replace scarce labor, reducing labor costs, improving productivity, and strengthening the capacity of agricultural systems to cope with external shocks. This process also contributes to rural economic growth and improved agricultural production efficiency, further supporting agri-food system resilience. Based on the above analysis, this study proposes the following hypothesis:

*H2c*: *The promotion and adoption of modern agricultural technology enhances agri-food system resilience by promoting the development of agricultural finance.*

Fourth, the promotion and adoption of modern agricultural technology can enhance agri-food system resilience by improving urban–rural coordination. One of the key obstacles to coordinated urban–rural development is the limited flow of production factors. From the perspective of capital competition theory, rural areas are at a clear disadvantage when competing with urban areas, leading to a greater concentration of resources in cities and non-agricultural sectors. This results in significant resource shortages in rural regions and restricts their development (74, 75). Rural areas face relatively weak infrastructure and increasingly severe aging problems, while urban–rural income inequality poses significant challenges to agricultural development (76). Therefore, addressing the resource disadvantages faced by rural areas is essential for promoting balanced urban–rural development (77).

The development of NMADZ and the promotion and adoption of modern agricultural technologies have reshaped the resource competition landscape between urban and rural areas. With the widespread adoption of new technologies, modern agricultural technology promotion and adoption is becoming a key force in advancing rural revitalization and urban–rural integration, offering new opportunities for building a development model where urban areas support rural progress and both share the benefits (78).

The promotion and adoption of modern agricultural technology contributes to urban–rural coordination by narrowing income gaps, promoting deeper integration of industrial chains, and improving agricultural productivity (79). Through access to information and communication technologies, farmers are better able to acquire knowledge about agricultural production and new technologies. This allows them to choose more efficient farming methods, techniques, and crop varieties, gradually improving productivity and thereby strengthening Agri-food system resilience. Based on the above analysis, this study proposes the following hypothesis:

*H2d*: *The promotion and adoption of modern agricultural technology enhances agri-food system resilience by improving urban–rural coordination.*

## 3 Research design

### 3.1 Data sources

This study employs panel data from 2,312 county-level administrative districts in China covering the period 2006–2023 to examine the impact and underlying mechanisms of the NMADZ policy on agri-food system resilience. The NMADZ represents a gradually implemented policy, which provides a favorable quasi-natural experimental setting for evaluating policy effects using a multi-period difference-in-differences approach. Specifically, a total of 283 counties were designated as pilot zones in three batches in 2010, 2012, and 2015, forming the intervention group, while counties without NMADZ designation served as the control group. Based on these data, we applied the multi-period difference-in-differences method to estimate the policy’s effects on agri-food system resilience.

Due to data availability constraints, counties with severe data deficiencies, such as Suixian and Hengnan, were excluded from the analysis. The data used in this study were primarily obtained from official and authoritative sources, including databases released by the National Bureau of Statistics of China, statistical reports from provincial bureaus, the *China Rural Statistical Yearbook*, and the *China County Statistical Yearbook*. To ensure data completeness and maintain research quality, missing values were scientifically and reasonably supplemented using interpolation methods.

### 3.2 Model specification

This study employs a multi-period DID model to empirically examine the impact of modern agricultural technology promotion and adoption on agri-food system resilience. The DID model, based on a quasi-natural experiment, effectively addresses estimation bias caused by endogeneity, thereby ensuring a more accurate identification of causal relationships. The multi-period DID approach is well suited to policies implemented in stages. It enables the observation of policy effects through empirical analysis, helps identify potential biases during the policy diffusion process, and allows for the examination of dynamic trends before and after the policy (80). Using the rollout of this policy starting in 2010 as an exogenous shock, and following the methodology of Li et al. (81), the multi-period DID model is constructed as shown in Equation (5):


(5) $$\mathrm{RES}I_{\mathrm{it}}=\alpha_{o}+\alpha_{1}\text{POLIC}Y_{\mathrm{it}}+\alpha X_{\mathrm{it}}^{'}+\mu_{i}+\nu_{t}+\varepsilon_{\mathrm{it}}$$

In the model, i represents the county, t represents the year, $\mathrm{RES}I_{\mathrm{it}}$ is the dependent variable, and $\text{POLIC}Y_{\mathrm{it}}$ is the independent variable used to capture the exogenous impact of the NMADZ policy. Specifically, $\text{POLIC}Y_{\mathrm{it}}={\text{treat}}_{i}\times {\text{post}}_{t}$ represents the interaction term of the NMADZ policy, where ${\text{treat}}_{i}$ is the dummy variable for the intervention group and ${\text{post}}_{t}$ is the dummy variable for time. If a county was included in the pilot list of the NMADZ policy in year t, then in year t and subsequent years $\text{POLIC}Y_{\mathrm{it}}=1$; otherwise, $\text{POLIC}Y_{\mathrm{it}}=0$. $X_{\mathrm{it}}^{'}$ is a vector of control variables, including income level, sown area, agricultural production foundation, level of agricultural modernization, grain production, and agricultural investment. $\mu_{i}$ denotes county fixed effects, $\nu_{t}$ denotes year fixed effects, and $\varepsilon_{\mathrm{it}}$ is the error term.

### 3.3 Variable definitions

#### 3.3.1 Dependent variable

Agri-food system resilience ($\mathrm{RES}I_{\mathrm{it}}$). Drawing on the counterfactual measurement approach, this study takes the state of the agricultural system on the eve of the 2008 global financial crisis as the ideal baseline. Agri-food system resilience is measured by the difference between the actual and expected growth rates of economic indicators such as agri-food output and employment (82, 83). Based on the static Verdoorn’s law, a spatial autoregressive model is constructed to examine the spatial dependence of agricultural employment across regions. The specific calculation formula is as follows:


(6) $$\ln x_{t}=\alpha_{1}+\mathrm{\rho W}\ln x_{t}+\gamma \ln x_{t-1}+\beta \ln y_{t}+\varepsilon_{t}$$

In the model, $x_{t}$ denotes the level of agricultural employment and is measured by the number of people engaged in farming, forestry, animal husbandry, and fishery, with data obtained from the National Bureau of Statistics of China. $y_{t}$ denotes the total level of agri-food output and is measured by the added value of the primary industry, which comprises agriculture, forestry, animal husbandry, and fishery, and the corresponding data are also sourced from the National Bureau of Statistics of China.$W\ln x_{t}$ captures the spatial lag effect of agricultural employment in neighboring regions, and $\varepsilon_{t}$ is the random disturbance term. Following the method of Doran and Fingleton (84), Equation 6 is estimated using the difference GMM approach. Furthermore, since the first batch of NMADZ was established in 2010, the year 2006 is selected as the baseline in order to establish a clear pre-policy window. Based on the actual agricultural output growth data of each county, the counterfactual output growth rate, which represents the rate that would have occurred in the absence of external shocks, is estimated (85). In addition, the counterfactual agricultural labor productivity, which refers to the productivity level in the absence of external shocks, is also inferred for each county.

Drawing on the discussion by Martin et al. (86) on economic resilience, this study defines agri-food system resilience as the extent to which the potential growth rate of agriculture can be maximized under external shocks. It is measured by the difference between the actual potential growth rate and the counterfactual potential growth rate. To calculate this, the study applies the HP filter to estimate both the actual and counterfactual potential growth rates, using their difference to assess agri-food system resilience across regions.

#### 3.3.2 Independent variable

The core independent variable is the interaction term $\text{POLIC}Y_{\mathrm{it}}$ representing the NMADZ policy. Treating the policy as a quasi-natural experiment, the core explanatory variable is expressed as the interaction ${\text{treat}}_{i}\times {\text{post}}_{t}$ between a dummy variable indicating whether a county is designated as a NMADZ and a dummy variable indicating the period of participation. If a county is included in the list of NMADZ in a given year, the variable takes the value of 1 for that year and the years that follow; otherwise, it takes the value of 0. The list of demonstration zones is obtained from the official website of the Ministry of Agriculture and Rural Affairs of China. Based on the release dates published by the Ministry, the years 2010, 2012, and 2015 correspond to the first, second, and third batches of designated pilot zones, respectively.

**Figure 3:**
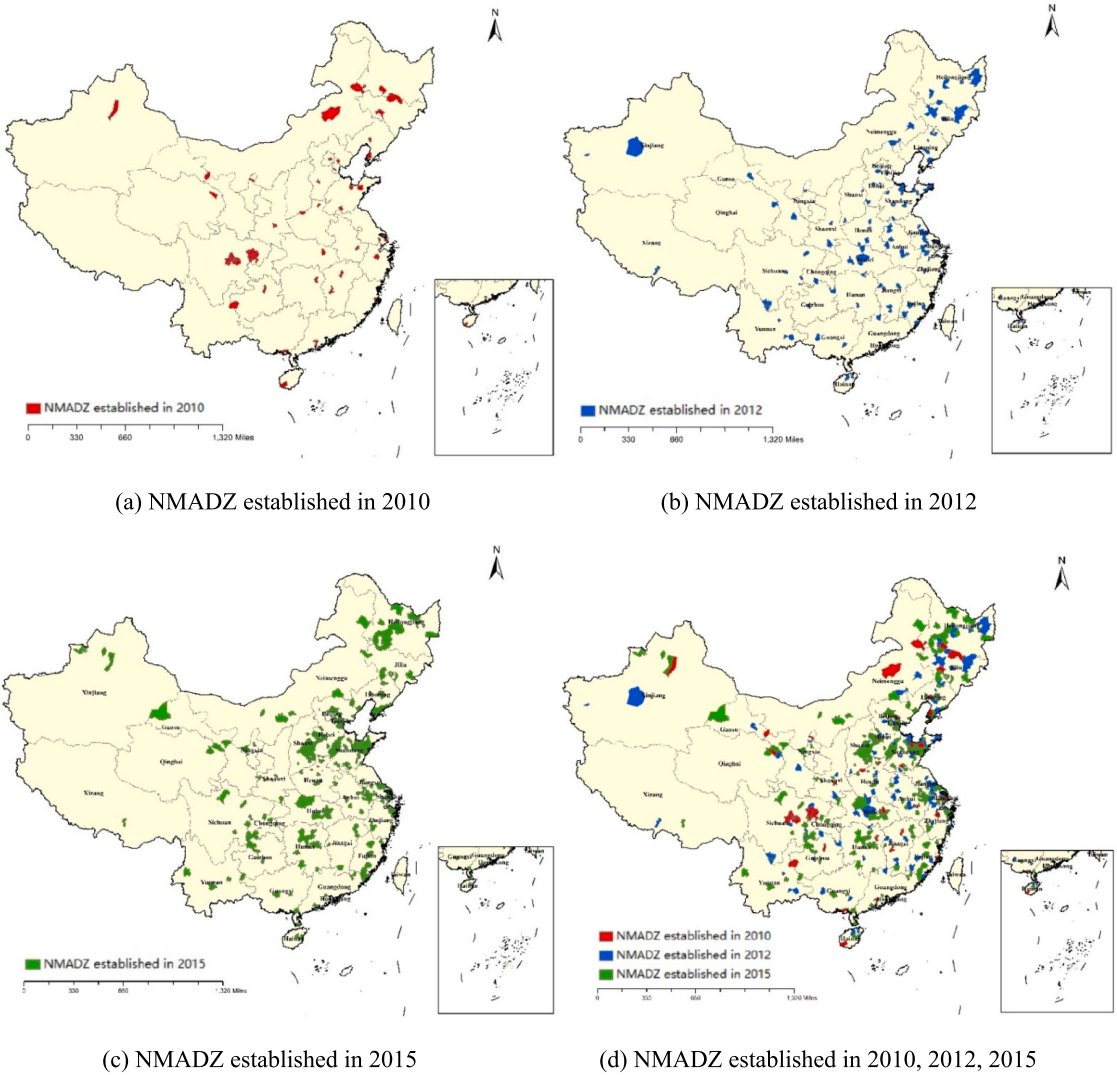
Spatiotemporal dynamics of the NMADZ. **(a)** NMADZ established in 2010. **(b)** NMADZ established in 2012. **(c)** NMADZ established in 2015. **(d)** NMADZ established in 2010, 2012, 2015.

The regional distribution of the first, second, and third batches of NMADZ is shown in Figure 3. As illustrated, the first batch of demonstration zones was relatively evenly distributed across provinces. Sichuan Province, which is the only major grain-producing region in western China, received a relatively large number of demonstration zones in the first batch, and these zones were more spatially clustered. In comparison, the second and third batches were mainly located in the eastern regions of China. It is important to note that the demonstration zones across the three batches exhibit strong spatial clustering. Most of the zones established in the second batch are adjacent to those from the first batch, and the third batch is largely concentrated near the zones from the previous two batches.

#### 3.3.3 Control variables

In addition to the policy’s impact on agri-food system resilience, it is necessary to control for other factors that may influence resilience. Compared with earlier studies that may have overlooked certain variables (87–89), this study enhances analytical rigor by controlling for potential influencing factors from multiple dimensions. In particular, we pay close attention to incorporating variables related to natural shocks and systematically control for a range of confounding factors that could simultaneously affect both policy implementation and outcomes, as described below:

1. Income level (lnincome). As rural living standards improve, demand for modern agricultural technologies and higher agri-food system resilience also increases. This is measured by the per capita disposable income of rural residents.
2. Sown area (lnsown-area). Sown area reflects the scale and structure of agricultural production and is measured by the total sown area of crops.
3. Agricultural production foundation (lnfoundation). Cultivated land is the fundamental resource for agricultural production. This is measured by the area of commonly used arable land (in hectares).
4. Level of agricultural modernization (lnmodernization). Facility agriculture, supported by technology, represents the direction of modern agricultural development. This is measured by the land area used for facility agriculture.
5. Grain production (lnproduction). Grain output reflects the level of agricultural development and supply capacity. The promotion and adoption of modern agricultural technologies can increase total grain production, thereby affecting agri-food system resilience. This is measured by total regional grain output.
6. Agricultural investment (lninvestment). Agricultural investment is an important support for agricultural modernization. This is measured by rural fixed asset investment.
7. Precipitation (lnprecipitation): Precipitation serves as a fundamental indicator for assessing extreme weather events and is measured by the average precipitation in each region.
8. Low temperature (lnlow-temperature): Low temperature is measured by the area affected, expressed in hectares, as a result of damage caused by low temperatures.
9. Wind and hail (lnwind-hail): Wind and hail are measured by the area affected, expressed in hectares, as a result of damage caused by wind and hail.

#### 3.3.4 Descriptive statistics of variables

Table 2 presents the descriptive statistics of the main variables. The mean represents the average level of each variable, the minimum shows the lower bound of the observed values, and the maximum indicates the upper bound. The results reveal that the mean of agri-food system resilience (RESI) is −0.5407, with a range from −22.2660 to 22.6986, suggesting that there is no pronounced disparity in agri-food system resilience across the country. The mean of the interaction term for the NMADZ policy (POLICY) is 0.1029, indicating that 10.29% of the sample was treated, and the proportion of treated observations to the control group is relatively balanced. The control variables also exhibit notable individual differences, implying that the sample as a whole has good discriminatory power. The descriptive statistical results of the variables are reported in Table 2.

**Table 2:** Variable definitions and descriptive statistics.

Variable	Obs.	Mean	Standard deviation	Minimum	Maximum
RESI	41,616	−0.5407	3.2404	−22.2660	22.6986
POLICY	41,616	0.1029	0.3039	0.0000	1.0000
lnincome	41,616	8.7201	0.6960	6.3733	10.6946
lnsown-area	41,616	3.6669	1.1327	0.0000	6.3891
lnfoundation	41,616	10.1164	1.2868	1.0986	13.1189
lnmodernization	41,616	5.9397	1.8514	0.6931	12.0952
lnproduction	41,616	11.6881	1.5142	0.0000	15.1077
lninvestment	41,616	12.6557	1.5614	1.6094	17.6117
lnprecipitation	41,616	0.0030	0.0015	0.0002	0.0088
lnlow-temperature	41,616	3.4659	1.7541	0.0953	6.5174
lnwind-hail	41,616	3.9819	1.4521	0.0000	6.7010

Table 3 presents the differences between the treatment group and the control group. Before the policy implementation, that is, during 2006–2009, the mean agri-food system resilience of the treatment group (T*_before_*) was −0.7007, and that of the control group (C*_before_*) was −0.7082. This indicates that prior to the implementation of the NMADZ policy, the agri-food system resilience of the treatment and control groups was nearly identical, demonstrating that the two groups were comparable and satisfying the parallel trend assumption of the DID approach. After the policy implementation, the mean agri-food system resilience of the treatment group (T*_after_*) and the control group (C*_after_*) both increased, but the increase was greater in the treatment group. The absolute difference between the two groups widened from 0.0075 before the policy to 0.0342 after the policy, suggesting that the implementation of the NMADZ policy exerted a positive impact on agri-food system resilience.

**Table 3:** Differences between the treatment group and the control group.

Type	Mean value		Policy-period difference	Overall mean
	2006–2009	2010–2023		
Treatment group	T*_before_* = −0.7007	T*_after_* = −0.4697	T*_before_* - T*_after_* = −0.2310	−0.432
Control group	C*_before_* = −0.7082	C*_after_* = −0.5039	C*_before_* - C*_after_* = −0.2043	−0.552
Difference (treatment-control)	T*_before_* - C*_before_* = 0.0075	T*_after_* - C*_after_* = 0.0342	Difference-in-differences test	−0.120^**^

Standard errors in parentheses *p < 0.1, **p < 0.05, ***p < 0.01.

## 4 Empirical analysis

### 4.1 Baseline regression

Based on model (1), the empirical analysis examines the impact of modern agricultural technology promotion and adoption on agri-food system resilience, and the benchmark regression results are reported in Table 4. Specifically, without controlling for related variables, the estimated coefficient of the interaction term for the NMADZ policy is 0.1887 at the 1% significance level, indicating that the promotion and adoption of modern agricultural technologies induced by this policy increased agri-food system resilience by 18.87%. After controlling for related variables, the regression coefficient of the interaction term is 0.1531 at the 1% significance level, showing that the policy-driven promotion and adoption of modern agricultural technologies enhanced agri-food system resilience by 15.31%. This result demonstrates that, compared with non-pilot counties, the promotion and adoption of modern agricultural technologies strengthened agri-food system resilience in the pilot counties. The finding is consistent with Hypothesis H1 and extends the perspective of existing research (90, 91). Moreover, it is supported by other studies (12, 92).

**Table 4:** Baseline regression results.

Variable	(1)	(2)
POLICY	0.1887^***^	0.1531^***^
	(0.0689)	(0.0529)
Constant	4.5630^***^	0.6814^***^
	(1.5606)	(0.0072)
Control variable	No	Yes
Year fixed effect	Yes	Yes
County fixed effect	Yes	Yes
*N*	41,616	41,616
*R* ^2^	0.232	0.394

Standard errors in parentheses *p < 0.1, **p < 0.05, ***p < 0.01.

### 4.2 Parallel trend test

**Figure 4:**
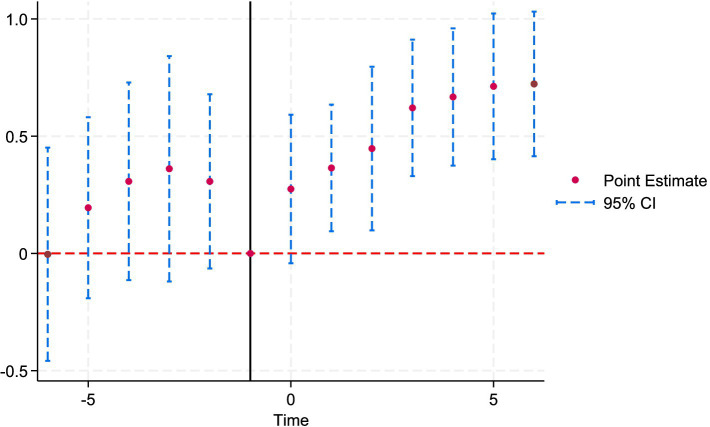
Results of the parallel trend test.

The parallel trend test is a key identifying assumption of the DID model. It requires that, in the absence of the NMADZ policy intervention, the treatment and control groups would exhibit the same trajectory in agri-food system resilience. Figure 4 presents the event-study coefficients for each period along with their 95% confidence intervals. In terms of statistical significance, the post-treatment coefficients are positive and significant, indicating that the policy shock exerts a measurable effect on the promotion and adoption of regional agricultural enterprise activity and thus satisfies the parallel-trends assumption. Moreover, the coefficients display an overall upward trajectory, suggesting that the policy’s impact on agri-food system resilience is both positive and sustainable.

Under the policy context of establishing NMADZ, increases in farmers’ income lead to greater consumption of higher-quality food, reflecting an optimization of the consumption structure. A rise in the Engel coefficient indicates enhanced stability of food consumption, which in turn reflects an improvement in farmers’ risk resilience. Through the dynamic regression results of the Engel coefficient, we examine the policy’s impact on rural household consumption structure and the resilience of the agricultural economy. Table 5 shows that all coefficients in the pre-treatment period are statistically insignificant, suggesting that there was no difference in the trend of Engel coefficients between the treatment and control groups before the policy was implemented. This finding satisfies the parallel trends assumption of the DID model and supports the causal interpretation of the policy effect. The coefficient in the year of policy implementation is 0.2745 and is not significant. The coefficients for lag 1 and lag 2 increase significantly, indicating that the policy effect exhibits a one- to two-year delay, which may be related to the gradual implementation of agricultural technology diffusion and industrial chain integration. The coefficients for lag 3 through lag 6 further increase to the range of 0.6205 to 0.7225, with results being statistically significant. This pattern demonstrates that the policy effect accumulates over time, which is consistent with the theoretical expectation that NMADZ gradually enhance agri-food system resilience.

**Table 5:** Parallel trends test and analysis of policy dynamic effects.

Variable	Engel coefficient
	(1)
Lead 6	−0.0035
	(0.2260)
Lead 5	0.1945
	(0.1960)
Lead 4	0.3070
	(0.2140)
Lead 3	0.3605
	(0.2440)
Lead 2	0.3070
	(0.1885)
Lag 0	0.2745^*^
	(0.1605)
Lag 1	0.3640^***^
	(0.1370)
Lag 2	0.4470^**^
	(0.1770)
Lag 3	0.6205^***^
	(0.1180)
Lag 4	0.6670^***^
	(0.1485)
Lag 5	0.7125^***^
	(0.1575)
Lag 6	0.7225^***^
	(0.1565)
Constant	3.2210^***^
	(0.3377)
*N*	41,616
*R* ^2^	0.910

Standard errors in parentheses *p < 0.1, **p < 0.05, ***p < 0.01.

**Figure 5:**
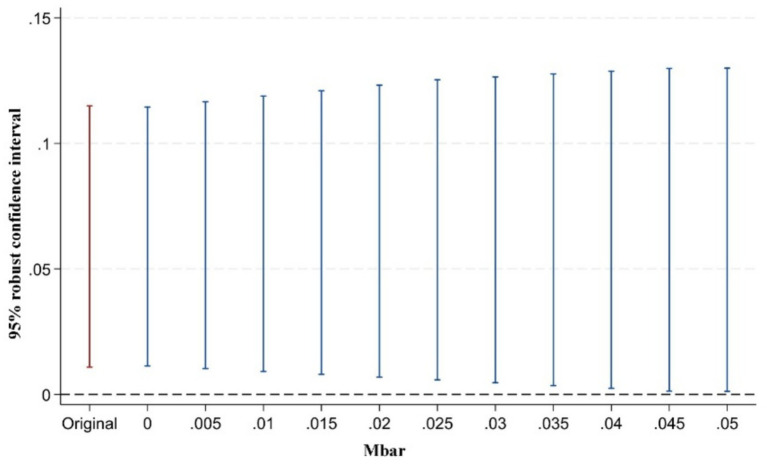
Sensitivity test of the parallel trends assumption in the second year after policy implementation.

**Figure 6:**
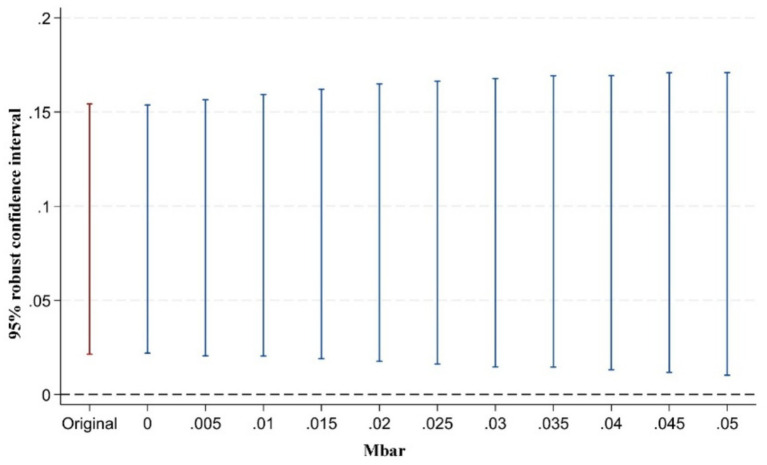
Sensitivity test of the parallel trends assumption in the third year after policy implementation.

**Figure 7:**
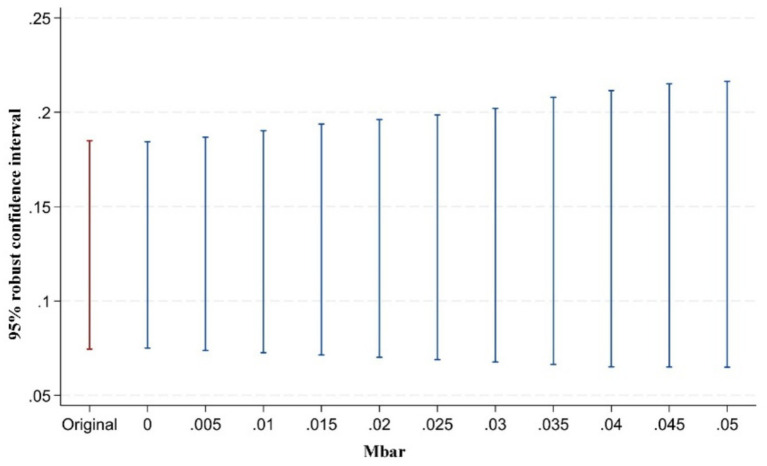
Sensitivity test of the parallel trends assumption in the fourth year after policy implementation.

However, some scholars have argued that pre-treatment trend tests cannot serve as valid empirical evidence for the parallel trends assumption (93). Traditional pre-treatment trend tests are statistically low-powered and may introduce bias and distortion in estimation and inference. To address this issue, Rambachan and Roth (94) proposed a testing approach for situations where the parallel trends assumption may be violated. The key idea is to conduct inference and sensitivity analysis on the confidence intervals of post-treatment point estimates. The test consists of two parts: first, constructing the maximum deviation from parallel trends (Mbar); second, constructing the confidence intervals of post-treatment point estimates corresponding to this degree of deviation. If the confidence interval of the post-treatment estimates does not include zero under the maximum deviation, it indicates that the treatment effect is robust to violations of the parallel trends assumption. Following the approach of Biasi and Sarsons (95), this study sets the maximum deviation Mbar = 1 × standard error to examine the sensitivity of the treatment effect to the parallel trends assumption after the implementation of the pilot policy. This approach to testing the sensitivity of the parallel trend assumption is still rarely applied in the existing literature. Our study presents in Figures 5–7 the results of parallel trend sensitivity tests for the treatment effects of the second, third, and fourth phases of policy implementation under constraints on relative deviations. This practice greatly enhances the rigor and transparency of the research and provides strong evidence that, within the range of relative deviations, the positive effect of the NMADZ policy on agricultural resilience after its implementation is highly robust and successfully passes the parallel trend sensitivity test.

### 4.3 Test of heterogeneous treatment effects

When the treatment and control groups satisfy the parallel trend assumption, the two-way fixed effects model is a commonly used method for evaluating policy effects. However, recent studies indicate that, in order to obtain an unbiased estimate of the average treatment effect, the estimated coefficient of the interaction term (POLICY) for the NMADZ policy must also meet an additional condition beyond the parallel trend assumption, namely that the treatment effect remains constant both across groups and over time (96, 97). The impact of modern agricultural technology promotion and adoption on agri-food system resilience may vary across regions and periods, and the estimated coefficient of the interaction term (POLICY) for the “Later vs. Earlier Treated” comparison could introduce bias in the policy evaluation. To address this concern, this study first applies the Bacon decomposition method to test for the presence of negative weight problems. The results show that the proportion of negative weights from “Later vs. Earlier Treated” comparisons is only 2.1% (see Table 6). This suggests that the risk of bias when using a two-way fixed effects model to estimate the effect of modern agricultural technology promotion and adoption on agri-food system resilience is low, and the conclusions are robust.

**Table 6:** Bacon decomposition.

POLICY	0.168

Type	Weights	Estimated coefficients
Earlier vs. Later treated	0.012	0.167
Later vs. Earlier treated	0.021	0.006
Treated vs. Never treated	0.967	0.174

In the Bacon decomposition, control variables are not included (consistent with the policy’s implementation level), and county and year fixed effects are employed.

### 4.4 Placebo test

**Figure 8:**
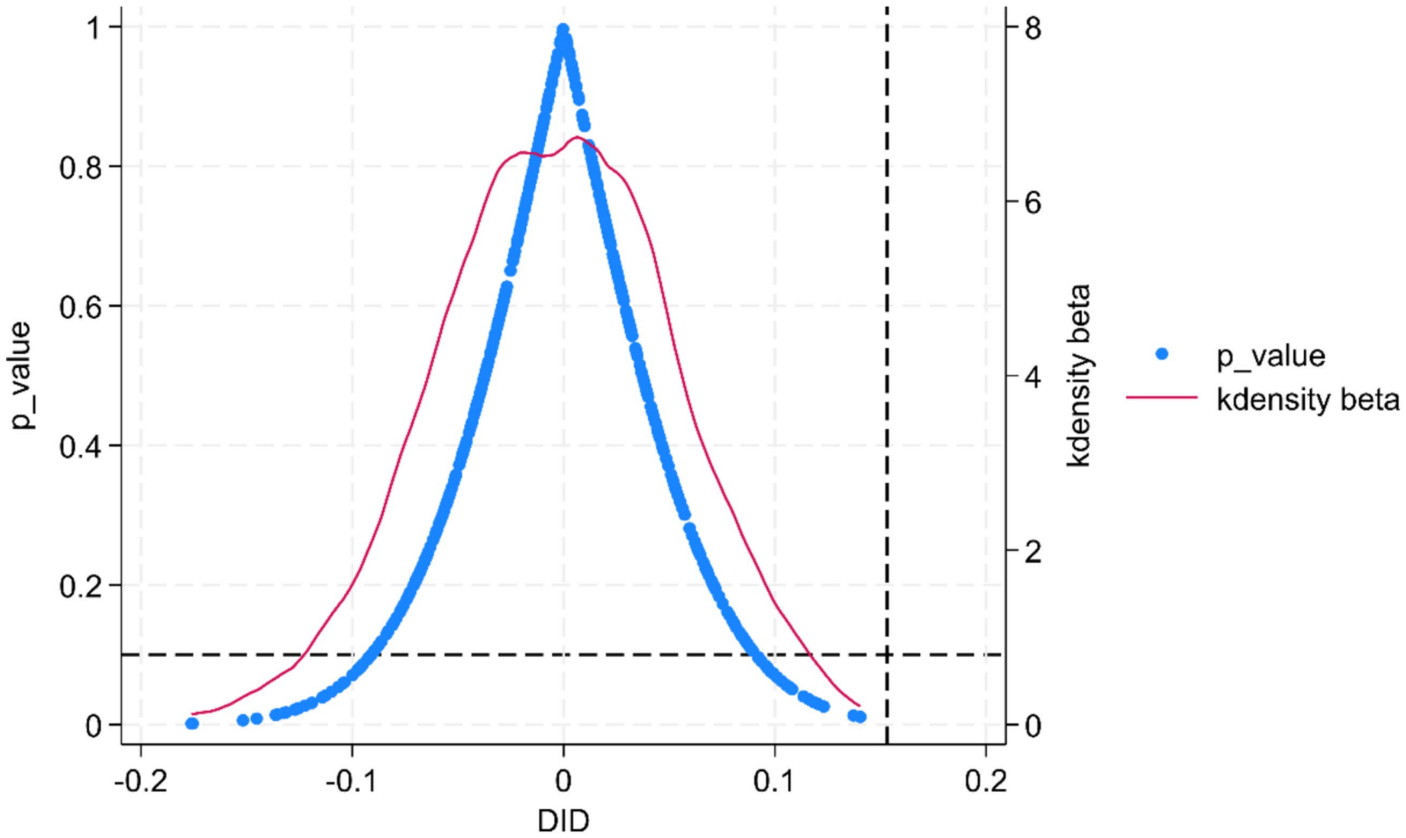
Placebo test results.

To eliminate the influence of confounding factors, we conducted a robustness check using a placebo test. Drawing on the distributional characteristics of the DID variable, we randomly assigned treatment status and performed 500 simulated placebo iterations of the above DID analysis to demonstrate that our results are not driven by chance. The findings, presented in Figure 8, show the distributions of the estimated coefficients and their *p*-values for the randomly generated treatment groups. The simulated coefficients closely follow a normal distribution, fluctuate around zero, and lie well away from the baseline estimate. This indicates that our identified policy effect is not materially influenced by unobserved factors, offering further support for the conclusion that the pilot policy enhances agri-food system resilience.

### 4.5 PSM-DID

Since the selection of counties for the NMADZ policy was not random, this study adopts propensity score matching combined with difference-in-differences (PSM-DID) and propensity score matching with inverse probability weighting and difference-in-differences (PSM-IPW-DID) to mitigate potential endogeneity problems arising from self-selection bias and omitted variables in the pilot county selection process. These methods ensure that, after matching, there are no significant differences between the treatment and control groups. By reasonably controlling for differences between pilot and non-pilot counties, the tests are conducted, and the corresponding regression results are presented in Table 7. The results show that under both the PSM-DID and PSM-IPW-DID methods, the estimated coefficient of the interaction term (POLICY) for the NMADZ policy remains significantly positive, providing further confirmation of the reliability of the estimation results.

**Table 7:** PSM-DID and PSM-IPW-DID regression results.

Variable	PSM-DID	PSM-IPW-DID
	(1)	(2)
POLICY	0.1543^**^	0.1782^***^
	(0.0697)	(0.0338)
Constant	0.5854^***^	0.4008^***^
	(0.0342)	(0.0578)
Control variable	Yes	Yes
Year fixed effect	Yes	Yes
County fixed effect	Yes	Yes
*N*	36,698	36,718
*R* ^2^	0.327	0.303

Standard errors in parentheses *p < 0.1, **p < 0.05, ***p < 0.01.

### 4.6 Endogeneity test

To mitigate the potential endogeneity problem between NMADZ and agri-food system resilience, we construct a second instrumental variable by interacting county-level terrain flatness with the national average mechanization level per mu. Terrain, as a natural endowment, remains essentially unchanged over time and lacks dynamic variation, whereas the national average mechanization level per mu has steadily increased, reflecting technological progress at the national level. By interacting these two variables, the instrumental variable incorporates a macro-level temporal trend.

The validity of this construction rests on two key considerations. First, relevance: the establishment of NMADZ requires favorable natural terrain conditions. Counties with flatter terrain face lower transportation and maintenance costs, achieve higher efficiency in mechanized operations, and are thus more likely to adopt and promote technologies, thereby satisfying the relevance condition of the instrumental variable. Second, exogeneity: county-level terrain flatness is determined by natural geographic endowments and remains stable over time, unaffected by policies or economic activities. The national average mechanization level per mu represents the macro-level trend of technological diffusion. After controlling for county fixed effects and year fixed effects, the interaction term does not directly affect the agri-food system resilience but influences it only through the adoption of modern agricultural technologies. Consequently, the instrumental variable is not systematically correlated with the error term of the dependent variable, thereby meeting the exogeneity requirement. Table 8 reports the instrumental variable estimation results obtained using the two-stage least squares (2SLS) method. The estimated coefficient of the interaction term (POLICY) for the NMADZ policy is significantly positive, which is consistent with the benchmark regression.

**Table 8:** Instrumental variable estimation.

Variable	(1)
POLICY	0.8942^***^
	(0.0417)
Control variable	Yes
Year fixed effect	Yes
County fixed effect	Yes
*N*	41,616
Kleibergen-Paap rk LM statistic	71.99
Kleibergen-Paap rk Wald F statistic	64.30

Standard errors in parentheses *p < 0.1, **p < 0.05, ***p < 0.01.

### 4.7 Additional robustness checks

This study verifies the robustness of the benchmark regression results through a series of tests, including changing the time window, changing the fixed effects structure, controlling for the impact of other pilot policies, applying quantile regression, and performing winsorization. The findings indicate that after the implementation of the NMADZ policy, the promotion and adoption of modern agricultural technologies exert a positive effect on agri-food system resilience. Due to space limitations, the detailed procedures and results of the other robustness checks are presented in the appendix of the Supplementary material, including Appendix Tables A1, A2.

## 5 Further analysis

### 5.1 Mechanism test

As discussed in the previous theoretical analysis, the promotion and adoption of modern agricultural technology may enhance agri-food system resilience through several channels: strengthening agricultural technological innovation capacity, increasing local government attention to agriculture, promoting agricultural financial development, and improving urban–rural coordination.

#### 5.1.1 Enhancing agricultural technological innovation capacity

To measure agricultural Technological innovation capacity, this study uses the number of granted valid agricultural invention patents (98–100). The regression results are reported in column (1) of Table 9. The estimated coefficient of the interaction term (POLICY) for the NMADZ policy is significantly positive, indicating that the rapid development of modern agriculture enhances agricultural technological innovation capacity, facilitates the promotion and adoption of modern agricultural technologies, and strengthens agri-food system resilience. Taken together, these findings support Hypothesis H2a. Our conclusion is further supported by Wan et al. (101).

**Table 9:** Mechanism test results.

Variable	Agricultural technological innovation capacity	Local government attention to agriculture	Agricultural financial development	Urban–rural coordination
	(1)	(2)	(3)	(4)
POLICY	0.2702^***^	0.2083^***^	1.3196^***^	2.8759^***^
	(0.0180)	(0.0101)	(0.0081)	(0.1304)
Constant	1.4788^***^	2.2001^***^	3.7006^***^	0.4092^***^
	(0.3012)	(0.1690)	(0.1404)	(0.0212)
Control variable	Yes	Yes	Yes	Yes
Year fixed effect	Yes	Yes	Yes	Yes
County fixed effect	Yes	Yes	Yes	Yes
*N*	41,616	41,616	41,616	41,616
*R* ^2^	0.161	0.103	0.275	0.146

Standard errors in parentheses *p < 0.1, **p < 0.05, ***p < 0.01.

#### 5.1.2 Increasing local government attention to agriculture

When a region places greater importance on agricultural development, it is more likely to take proactive steps to improve agri-food system resilience, including implementing relevant policies. This study uses a word frequency analysis to measure the level of attention local governments give to agriculture. Based on the concept and connotations of agri-food system resilience and drawing on various policy documents and media reports, this study selects keywords such as “agriculture” and “agricultural modernization” and applies textual analysis to regional government documents to perform term-frequency analysis. The natural logarithm of each search term’s occurrence count is then used as an indicator of local governments’ attention to agriculture (102, 103). The regression results are reported in column (2) of Table 9. The estimated coefficient of the interaction term (POLICY) for the NMADZ policy is significantly positive at the 1% level, indicating that the promotion and adoption of modern agricultural technologies increased local government attention to agriculture and in turn enhanced agri-food system resilience. Taken together, these findings support Hypothesis H2b. Furthermore, within the scope of our literature search, we did not identify prior studies examining the interaction between local government attention to agriculture and agri-food system resilience. Existing research has primarily applied government attention measures to studies of climate impacts on agricultural development (104, 105). By innovatively applying the concept of local government attention to agriculture in the context of modern agricultural technology promotion and adoption, this study demonstrates that the diffusion of modern agricultural technologies can raise local government attention to agriculture and thereby improve agri-food system resilience.

#### 5.1.3 Promoting agricultural financial development

The diffusion of modern agricultural technologies not only directly improves agricultural production efficiency but also indirectly strengthens the agri-food system resilience by promoting the development of agricultural finance. By reducing agricultural risks, modern technologies increase the willingness of financial institutions to extend credit, broaden financing channels, and enhance the provision of financial services. The rapid development of agricultural finance further underscores the level of government attention devoted to agriculture. To measure the development of agricultural finance, this study uses fiscal expenditures on agriculture, forestry, and water as a proxy (106, 107). The regression results are reported in column (3) of Table 9. The estimated coefficient of the interaction term (POLICY) for the NMADZ policy is significantly positive, indicating that the promotion and adoption of modern agricultural technologies facilitate the development of agricultural finance, thereby improving agri-food system resilience. This finding supports Hypothesis H2c and provides a new perspective beyond the work of Yang et al. (108). Yang et al. (108) argue that the development of financial technology can enhance the resilience of the agricultural economy, with agricultural technological innovation playing a critical mediating role. Our research places greater emphasis on agri-food system resilience. Unlike studies that focus on agricultural financial resilience, the present study addresses a broader concept of agri-food system resilience, which encompasses a wider range of influencing factors. We highlight the impact of the promotion and adoption of modern agricultural technologies on agri-food system resilience and also consider the positive contribution of agricultural financial development to resilience. The key difference from Yang et al. (108) lies in our empirical confirmation that the promotion and adoption of modern agricultural technologies can improve agri-food system resilience by fostering agricultural financial development. This difference arises because, with rapid social and economic progress, the promotion and adoption of modern agricultural technologies have become indispensable elements of agricultural development, and our study places particular emphasis on these factors.

#### 5.1.4 Improving urban–rural coordination

Urban–rural integration has been extensively examined in the literature (78, 109), but it has rarely been applied as an influencing factor in studies of agri-food system resilience. To measure the level of urban–rural coordination, this study uses the urban–rural dual structure coefficient as an indicator (110, 111). The regression results are reported in column (4) of Table 9. The estimated coefficient of the interaction term (POLICY) for the NMADZ policy is significantly positive, indicating that the promotion and adoption of modern agricultural technologies foster urban–rural coordination and thereby enhance agri-food system resilience. Taken together, these findings support Hypothesis H2d.

### 5.2 Heterogeneity analysis

To further investigate whether the impact of modern agricultural technology promotion and adoption on agricultural technology exhibits heterogeneity, this study examines five perspectives: regional transfer of rural labor, major grain-producing regions, policy implementation batches, ethnic minority concentration, and differences in educational attainment. Specifically, the analysis divides rural labor transfer into rural labor inflow and rural labor outflow, and also distinguishes between major and non-major grain-producing regions, the first, second, and third batches of NMADZ policy implementation, Han-majority and ethnic minority regions, as well as areas with higher and lower educational attainment. The regression results are presented in Table 10. Through this comprehensive heterogeneity analysis, we identify key boundary conditions under which the NMADZ policy operates, refine the previous overly generalized understanding of the policy’s effects, and propose more precisely targeted policy recommendations.

**Table 10:** Results of heterogeneity test 1.

Variable	Labor outflow	Labor inflow	Major grain-producing regions	Non-major grain-producing regions	The first batch	The second batch	The third batch
	(1)	(2)	(3)	(4)	(5)	(6)	(7)
POLICY	0.5515^***^	0.1275	0.2180^*^	0.2996^***^	0.0491	0.3933^**^	0.1050^**^
	(0.1955)	(0.0973)	(0.1239)	(0.1112)	(0.2818)	(0.1932)	(0.0499)
Constant	0.5686^***^	0.5340^***^	0.3030^***^	0.4148^***^	0.7309	0.1611^*^	0.1038^**^
	(0.0205)	(0.0195)	(0.0147)	(0.0322)	(0.5965)	(0.0839)	(0.0439)
Control variable	Yes	Yes	Yes	Yes	Yes	Yes	Yes
Year fixed effect	Yes	Yes	Yes	Yes	Yes	Yes	Yes
County fixed effect	Yes	Yes	Yes	Yes	Yes	Yes	Yes
*N*	15,171	25,374	13,564	27,115	851	1,659	4,936
*R* ^2^	0.114	0.150	0.427	0.423	0.127	0.131	0.137

Standard errors in parentheses *p < 0.1, **p < 0.05, ***p < 0.01.

#### 5.2.1 Heterogeneity analysis of regional labor transfer

The promotion and adoption of modern agricultural technology has accelerated rural labor outflow. This is because demonstration zones widely adopt technologies such as smart agricultural machinery and drones, which increasingly replace traditional manual labor, thereby reducing agriculture’s dependence on conventional labor. The promotion and adoption of modern agricultural technologies meets the practical needs of transitioning agricultural production toward modernization and increases reliance on mechanization-especially in major labor-exporting regions, where this demand is more pronounced. In areas with high levels of labor outflow, where agricultural labor is relatively scarce, improving land use and production efficiency becomes more critical. The promotion and adoption of modern agricultural technology can more effectively support labor substitution through mechanization, thereby strengthening agri-food system resilience. The estimated coefficient of the interaction term (POLICY) for the NMADZ policy is significant at the 1 percent level, as reported in columns (1) and (2) of Table 10, providing evidence in support of Hypothesis H1. Additionally, although modern agriculture improves efficiency, the profit margins in agriculture remain lower than those in the secondary and tertiary sectors, making it difficult to attract labor back to rural areas. Moreover, modern agricultural technology requires a higher skill level, which many traditional farmers or low-skilled migrant workers may not possess.

#### 5.2.2 Heterogeneity analysis of major grain-producing regions

Differences in agricultural functional zoning may affect the effectiveness of the NMADZ policy. Based on the National Medium- and Long-Term Plan for Food Security (2008–2020), this study divides the sample into major grain-producing regions and non-major grain-producing regions. As shown in Columns (3) and (4) of Table 10, the promotion and adoption of modern agricultural technology enhances agri-food system resilience in both types of regions. However, the effect is stronger in non- major grain-producing regions. On one hand, non- major grain-producing regions generally face resource disadvantages, and traditional agricultural models have weaker risk resistance. Modern agricultural technologies, through precise adaptation, can systematically compensate for these limitations. On the other hand, agricultural structures in non- major grain-producing regions tend to focus on higher-value-added crops and integrated operations, which are more compatible with industrial upgrading supported by modern technology. Given the convergence in policy standards and funding allocation, the demonstration zone policy is more effective in improving natural endowment constraints in non- major grain-producing regions.

#### 5.2.3 Heterogeneity analysis of implementation batches

After the implementation of the NMADZ policy, differences emerged in the effects of different batches on enhancing the agri-food system resilience. The estimated coefficient of the interaction term (POLICY) for the first batch is positive but not significant, whereas the second and third batches significantly improved agri-food system resilience at the 5% level, as shown in columns (5), (6), and (7) of Table 10. These differences are mainly due to phased optimization during the pilot and promotion stages, adjustments in resource allocation, and varying capacities of regions to adapt to policy implementation. The first batch of demonstration zones primarily focused on single-dimension investments in technology promotion and infrastructure construction. However, they lacked a systematic design for enhancing agri-food system resilience, and resource distribution was relatively coarse. In addition, due to the absence of mature technology adaptation standards and resilience evaluation systems, some modern agricultural technologies underperformed in practice due to complexity and high maintenance costs. The early experiences of the first batch served as trial-and-error inputs, providing valuable data and insights for subsequent improvements. Over time, resource allocation shifted from basic input toward more scientific investment, and regional strategies evolved into more diversified and adaptive models. Therefore, future policies should further strengthen dynamic evaluation and flexible adjustment, and establish additional NMADZ on the basis of the first, second, and third batches of NMADZ in order to address complex environmental challenges.

#### 5.2.4 Heterogeneity analysis of ethnic minority districts

China is a unified multi-ethnic country in which the Han nationality constitutes the majority, while ethnic minorities are distributed in a pattern characterized as “large-scale dispersal, small-scale concentration, and mixed inhabitation” (112). Ethnic minority districts are widely distributed, and the vast majority of them are located in frontier areas of China, ranging from Heilongjiang in the northeast to Xinjiang in the northwest, and to Tibet, Yunnan, and Guangxi in the southwest. Owing to their distinctive geographical locations, agricultural development in these districts has been relatively lagging (113). Consequently, differences between minority and Han-majority districts may influence the implementation outcomes of the NMADZ policy. After the establishment of demonstration zones, the improvement in agri-food system resilience differs between Han Chinese and minority districts. In Han Chinese districts, the policy exerts a significantly positive effect at the 5% level, indicating that the NMADZ policy has substantially enhanced agricultural resilience, as shown in column (1) of Table 11. This result can be explained by the fact that Han-majority districts generally possess more developed agricultural infrastructure and market networks, which enable them to absorb policy benefits more rapidly. Moreover, the promotion and adoption of modern agricultural technologies tend to favor standardized cultivation practices, which are more consistent with traditional Han farming methods. In contrast, minority districts exhibit a positive effect at the 10% level, suggesting that the policy has also enhanced agricultural resilience in these areas, although to a lesser extent than in Han Chinese districts, as reported in column (2) of Table 11. A possible explanation is that minority districts rely predominantly on traditional agriculture, which is less compatible with the industrialization-oriented objectives of the demonstration zone policy. In addition, language and cultural barriers in minority districts often delay the transmission of information, thereby weakening the effectiveness of policy outreach. For this reason, the promotion and adoption of modern agricultural technologies in minority districts should be adapted to local characteristics, with greater efforts devoted to establishing high-quality demonstration zones that are aligned with the distinctive features of these districts.

**Table 11:** Results of heterogeneity test 2.

Variable	Han Chinese districts	Ethnic minority districts	High education	Low education
	(1)	(2)	(3)	(4)
POLICY	0.6523^**^	0.1230^*^	0.1471^*^	0.1891
	(0.3057)	(0.0727)	(0.0802)	(0.4312)
Constant	4.6661	6.2401^***^	5.3792^***^	5.6133
	(2.3273)	(1.6191)	(1.4626)	(4.3459)
Control variable	Yes	Yes	Yes	Yes
Year fixed effect	Yes	Yes	Yes	Yes
County fixed effect	Yes	Yes	Yes	Yes
*N*	39,570	6,591	29,957	11,659
*R* ^2^	0.201	0.214	0.206	0.228

Standard errors in parentheses *p < 0.1, **p < 0.05, ***p < 0.01.

#### 5.2.5 Heterogeneity analysis of educational attainment

After the implementation of the NMADZ policy, differences in educational attainment appear to influence its effects on agri-food system resilience. Groups with higher levels of education are able to master modern technologies more quickly and make better use of the resources provided by the policy. In addition, these groups possess stronger agricultural risk management capabilities, allowing the policy’s impact to be amplified through the enhancement of human capital. As a result, higher educational attainment increases agricultural resilience at the 10% significance level, as shown in column (3) of Table 11, indicating that the policy is more effective in improving agricultural resilience in areas with higher educational levels. However, in areas with lower educational attainment, the estimated coefficient of the interaction term (POLICY) for the NMADZ policy is positive but not significant, as reported in column (4) of Table 11. This suggests that the policy’s impact is limited in these areas. A possible explanation is that groups with lower levels of education lack the capacity to apply modern technologies, making it difficult to achieve effective adoption and diffusion of agricultural innovations. Moreover, their limited access to information reduces participation in the policy, further constraining its effectiveness.

### 5.3 Three-dimensional kernel density analysis

Kernel density estimation provides a smoothed estimate of the probability density function of a random variable, capturing the distributional shape, characteristics, extensibility, and polarization trends. Compared with other estimation methods, kernel density estimation is less dependent on model assumptions and exhibits stronger robustness. In this section, we apply a Gaussian kernel function to estimate the kernel density and further analyze the spatiotemporal dynamics of agri-food system resilience in China.

**Figure 9:**
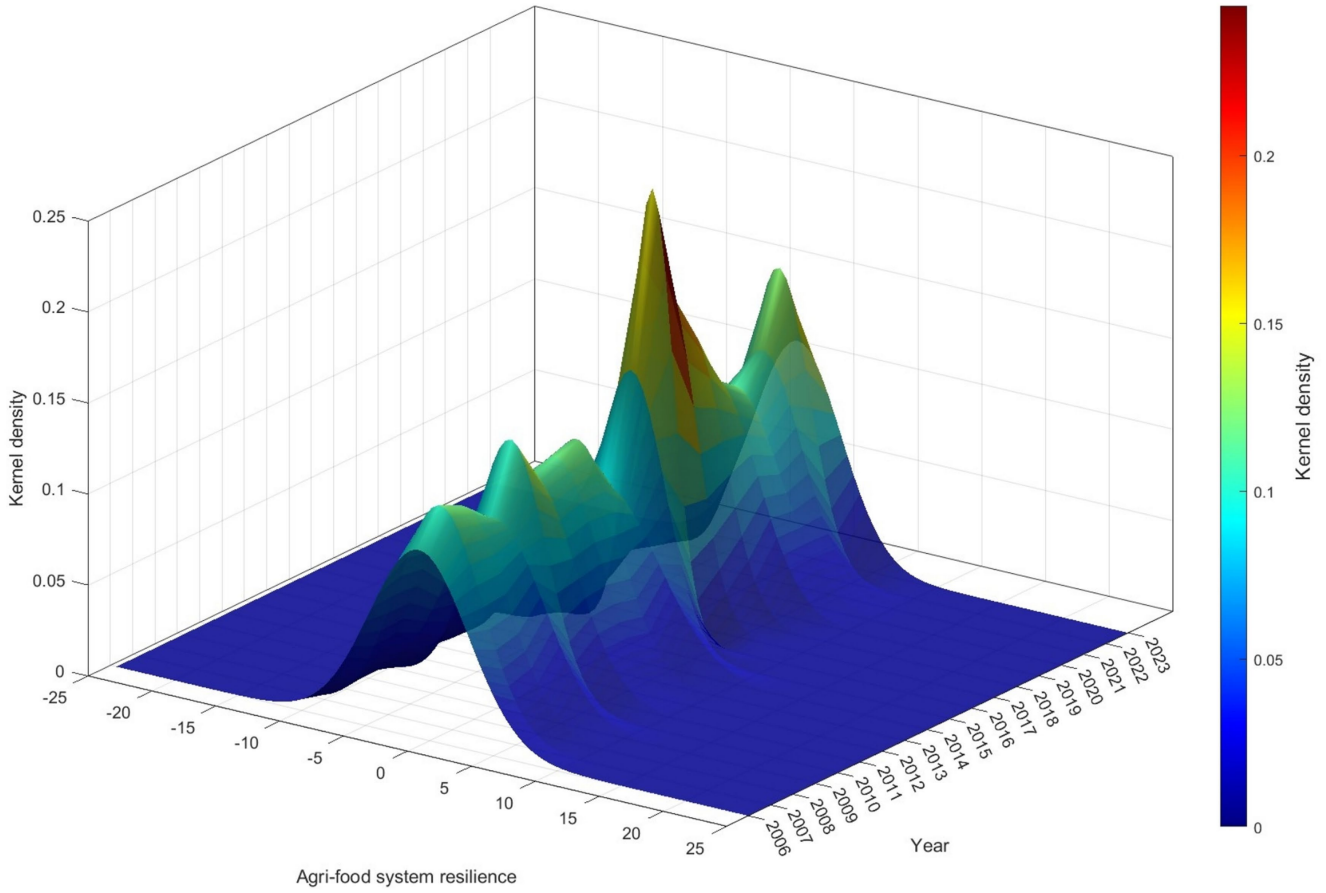
Three-dimensional kernel density analysis.

We construct a three-dimensional coordinate system integrating agri-food system resilience, time (year), and kernel density, and conduct an in-depth analysis of the spatiotemporal evolution of agri-food system resilience in China from 2006 to 2023, as illustrated in Figure 9. The X-axis represents agri-food system resilience, the Y-axis represents the year, and the Z-axis represents the kernel density. The spatial probability surface formed by the blue base and multicolored density peaks reveals the spatiotemporal variation in agri-food system resilience across different years.

Given that the first batch of NMADZ was launched in 2010, and the second batch was established in 2012, we observe a significant clustering effect of high-resilience regions emerging approximately 3 years after the initial policy implementation. In addition, the narrowing of the density peak width over time from 2006 to 2023 indicates a reduction in disparities in agri-food system resilience across counties, suggesting that the promotion and adoption of modern agricultural technologies have played a positive role in enhancing agri-food system resilience. This analysis provides a spatial measurement foundation for understanding how the adoption of modern agricultural technologies contributes to strengthening Agri-food system resilience.

## 6 Conclusion and policy implications

### 6.1 Conclusion

China is transitioning from a major agricultural country to a strong agricultural power, and the promotion and adoption of modern agricultural technologies play a pivotal role in enhancing agri-food system resilience and ensuring food security. The establishment of NMADZ and the large-scale adoption of modern agricultural technologies in China have provided valuable experience and managerial insights for developing countries. At the theoretical level, this study constructs a strategic game model among counties to explore the positive impact of modern agricultural technology promotion and adoption on agri-food system resilience. This study uses data from 2,312 counties in China from 2006 to 2023 and treats the NMADZ policy as a quasi-natural experiment. A multi-period DID approach is employed to empirically examine the impact of modern agricultural technology promotion and adoption on agri-food system resilience.

The findings show that the promotion and adoption of modern agricultural technologies significantly improves agri-food system resilience, and this conclusion remains robust across a series of validation tests. Mechanism tests suggest that the enhancement of agri-food system resilience occurs through several channels, including strengthening agricultural innovation capacity, increasing local government attention to agriculture, promoting agricultural financial development, and improving urban–rural coordination. The heterogeneity analysis shows that the diffusion of modern agricultural technologies has a more pronounced effect in counties experiencing labor outflow. Compared with major grain-producing regions, the positive impact is more evident in non-grain-producing areas. Similarly, relative to the first batch of pilot counties under the NMADZ policy, the promotion effect on agri-food system resilience is stronger in the second and third batches of pilot counties. In addition, the diffusion of modern agricultural technologies exerts greater positive effects in Han Chinese districts and in counties with higher levels of educational attainment. The kernel density analysis further indicates that the diffusion of modern agricultural technologies has reduced disparities in agri-food system resilience across counties.

### 6.2 Policy implications

Based on the above findings, several key insights can be drawn to enhance agri-food system resilience and ensure food security in developing countries:

First, establishing NMADZ and promoting the application of modern agricultural technologies can effectively improve agri-food system resilience. It is essential to continuously refine the top-level institutional design of these demonstration zones, sustain the implementation of relevant policies, and provide consistent policy support. The study finds that the pilot effects of the second and third batches of demonstration zones are significantly stronger than those of the first batch. Therefore, the scope of the NMADZ should be expanded and their implementation further institutionalized. Moreover, since the impact of modern agricultural technology promotion and adoption in ethnic minority districts has been less pronounced than in Han Chinese districts, the development of demonstration zones should take into account the distinctive characteristics of minority districts and establish models that align with local conditions.

Second, local governments should increase their attention to agriculture by strengthening the role of agricultural technology, improving the effectiveness of labor substitution through mechanization, actively investing in farmland infrastructure, and enhancing the quality of local agricultural services. They should also encourage the development of region-specific agricultural products. During the process of promoting and adopting modern agricultural technologies, particular consideration should be given to the barriers faced by women farmers who remain in rural areas. Professional skill training should be provided to them, along with access to channels that can improve agricultural productivity. In addition, more efficient management mechanisms should be established to improve policy implementation in agricultural product marketing and service delivery. Efforts should be made to enhance agricultural technology training and extension services, and to provide online technical support through e-commerce platforms to help farmers adopt advanced technologies.

Third, it is essential to vigorously promote the development of rural finance and to optimize urban–rural coordination. On the one hand, relevant policies should be formulated to encourage financial institutions to collaborate with enterprises along the agricultural industrial chain by providing comprehensive financial support and service guarantees. The widespread use of advanced financial instruments, such as those enabled by the digital economy, can further support the growth of rural finance. In addition, locally tailored financial tools should be designed to meet regional needs and to mitigate the financial risks that may constrain agricultural development. On the other hand, efforts should be made to strengthen the interconnection of urban and rural infrastructure, promote the joint construction and sharing of public services, and foster coordinated industrial development, thereby narrowing the urban–rural gap. Encouraging the flow of urban capital, technology, and talent into rural areas will further advance integrated urban–rural development.

### 6.3 Limitations and future research

Although this study demonstrates that the diffusion of modern agricultural technologies can significantly enhance the agri-food system resilience and provides valuable insights, certain limitations remain. The data used in this study are confined to 2,312 counties in China, without incorporating information from other developing countries. This may limit the generalizability of the conclusions, and further validation is needed regarding their applicability to other national or regional contexts. Future research could include cross-country or cross-regional datasets to explore how the effects of modern agricultural technology diffusion on agri-food system resilience vary across different settings. In addition, although we examined to the greatest extent possible whether heterogeneity exists in the effects of modern agricultural technology diffusion on resilience, some influencing factors, such as the number of left-behind women and the age structure of farmers, were not analyzed in depth because of limitations in data availability. Future studies may benefit from collecting more diverse datasets on factors influencing agri-food system resilience and from investigating how the impact of modern agricultural technology diffusion varies under these different conditions.

## Data Availability

The original contributions presented in the study are included in the article/Supplementary material, further inquiries can be directed to the corresponding author.

## References

[1] Kc U Campbell-Ross H Godde C Friedman R Lim-Camacho L Crimp S. A systematic review of the evolution of food system resilience assessment. Glob Food Secur. (2024) 40:100744. doi: 10.1016/j.gfs.2024.100744

[2] Benitez-Alfonso Y Soanes BK Zimba S Sinanaj B German L Sharma V. Enhancing climate change resilience in agricultural crops. Curr Biol. (2023) 33:1246–61. doi: 10.1016/j.cub.2023.10.028 38052178

[3] Arndt M Helming K. Agricultural diversification across spatial levels - a contribution to resilience and sustainability? Agric Ecosyst Environ. (2025) 385:109547. doi: 10.1016/j.agee.2025.109547,

[4] Ye LM Xiong W Li ZG Yang P Wu WB Yang GX. Climate change impact on China food security in 2050. Agron Sustain Dev. (2013) 33:363–74. doi: 10.1007/s13593-012-0102-0

[5] Stone J Rahimifard S. Resilience in agri-food supply chains: a critical analysis of the literature and synthesis of a novel framework. Supply Chain Manag Int J. (2018) 23:207–38. doi: 10.1108/SCM-06-2017-0201

[6] Oluwole O Ibidapo O Arowosola T Raji F Zandonadi RP Alasqah I. Sustainable transformation agenda for enhanced global food and nutrition security: a narrative review. Front Nutr. (2023) 10:1226538. doi: 10.3389/fnut.2023.1226538, PMID: 37599683 PMC10433737

[7] Belhadi A Kamble SS Mani V Benkhati I Touriki FE. An ensemble machine learning approach for forecasting credit risk of agricultural SMEs’ investments in agriculture 4.0 through supply chain finance. Ann Oper Res. (2025) 345:779–807. doi: 10.1007/s10479-021-04366-9, PMID: 34776573 PMC8576317

[8] Gomez M Grady C. A balancing act: the interplay of food supply chain resilience and environmental sustainability in American cities. Environ Res Lett. (2023) 28:124022. doi: 10.1088/1748-9326/ad0608

[9] Ingram J Bellotti W Brklacich M Achterbosch T Balázs B Banse M. Further concepts and approaches for enhancing food system resilience. Nat Food. (2025) 6:412. doi: 10.1038/s43016-025-01159-2 40102667

[10] Wood A Queiroz C Deutsch L González-Mon B Jonell M Pereira L. Reframing the local-global food systems debate through a resilience lens. Nature Food. (2023) 4:22–9. doi: 10.1038/s43016-022-00662-0, PMID: 37118580

[11] Lin JB Fan YC. Seeking sustainable performance through organizational resilience: examining the role of supply chain integration and digital technology usage. Technol Forecast Soc Change. (2024) 198:123026. doi: 10.1016/j.techfore.2023.123026

[12] Reggiani A. Network resilience for transport security: some methodological considerations. Transp Policy. (2013) 28:63–8. doi: 10.1016/j.tranpol.2012.09.007

[13] Martin R. Regional economic resilience, hysteresis and recessionary shocks. J Econ Geogr. (2012) 12:1–32. doi: 10.1093/jeg/lbr019

[14] Fabinyi M. Social-ecological systems, social diversity, and power: insights from anthropology and political ecology. Ecol Soc. (2014) 19:28. doi: 10.5751/ES-07029-190428

[15] Olsson P Galaz V Boonstra WJ. Sustainability transformations: a resilience perspective. Ecol Soc. (2014) 19:1. doi: 10.5751/ES-06799-190401

[16] Speranza CI Wiesmann U Rist S. An indicator framework for assessing livelihood resilience in the context of social–ecological dynamics. Glob Environ Change. (2014) 28:109–19. doi: 10.1016/j.gloenvcha.2014.06.005

[17] Troell M Naylor RL Metian M Beveridge M Tyedmers PH Folke C. Does aquaculture add resilience to the global food system? Proc Natl Acad Sci USA. (2014) 111:13257–63. doi: 10.1073/pnas.1404067111, PMID: 25136111 PMC4169979

[18] Altieri MA Nicholls CI Henao A Lana MA. Agroecology and the design of climate change-resilient farming systems. Agron Sustain Dev. (2015) 35:869–90. doi: 10.1007/s13593-015-0285-2

[19] Stone TF Huckins EL Hornbuckle EC Thompson JR Dentzman K. Equity and resilience in local urban food systems: a case study. Agric Hum Values. (2024) 41:1239–56. doi: 10.1007/s10460-024-10551-w

[20] Zurek M Ingram J Bellamy AS Goold C Lyon C Alexander P. Food system resilience: concepts, issues, and challenges. Annu Rev Environ Resour. (2022) 47:511–34. doi: 10.1146/annurev-environ-112320-050744

[21] Coopmans I Bijttebier J Marchand F Mathijs E Messely L Rogge E. COVID-19 impacts on Flemish food supply chains and lessons for Agri-food system resilience. Agric Syst. (2021) 190:103136. doi: 10.1016/j.agsy.2021.103136

[22] Rathi A. Is agrarian resilience limited to agriculture? Investigating the “farm” and “non-farm” processes of agriculture resilience in the rural. J Rural Stud. (2022) 93:155–64. doi: 10.1016/j.jrurstud.2019.12.015

[23] Volkov A Morkunas M Balezentis T Streimikiene D. Are agricultural sustainability and resilience complementary notions? Evidence from the north European agriculture. Land Use Policy. (2022) 112:105791. doi: 10.1016/j.landusepol.2021.105791

[24] Li B Gao YT. Impact and transmission mechanism of digital economy on agricultural energy carbon emission reduction. Int Rev Econ Finance. (2024) 95:103457. doi: 10.1016/j.iref.2024.103457

[25] Li KH Wang LP Wang LH. Consumption as the catalyst: analyzing rural power infrastructure and agricultural growth through panel threshold regression and data-driven prediction. Appl Energy. (2024) 365:123301. doi: 10.1016/j.apenergy.2024.123301

[26] Dong Y Qi C Gui C Yang Y. Spatial spillover effects of digital infrastructure on food system resilience: an analysis incorporating threshold effects and spatial decay boundaries. Foods. (2025) 14:1484. doi: 10.3390/foods14091484, PMID: 40361566 PMC12071979

[27] Su KQ Wang RX Han Z Chen JC Deng XZ. Examining the path of urban–rural industry convergence and its impacts on farmers’ income growth: evidence from Xinjiang Uyghur autonomous region, China. Ann Oper Res. (2023). doi: 10.1007/s10479-023-05762-z

[28] Sinclair K Curtis A Mendham E Mitchell M. Can resilience thinking provide useful insights for those examining efforts to transform contemporary agriculture? Agric Hum Values. (2014) 31:371–84. doi: 10.1007/s10460-014-9488-4

[29] Giannakis E Bruggeman A. Regional disparities in economic resilience in the European Union across the urban-rural divide. Reg Stud. (2020) 54:1200–13. doi: 10.1080/00343404.2019.1698720

[30] Guo J Fan L Feng P Sun X Xue S. Response of vegetation evapotranspiration to landscape pattern changes in an arid region: a case study of the loess plateau. China. Catena. (2025) 252:108878. doi: 10.1016/j.catena.2025.108878

[31] Pretty J. Intensification for redesigned and sustainable agricultural systems. Science. (2018) 362:eaav0294. doi: 10.1126/science.aav0294, PMID: 30467142

[32] Blazquez-Soriano A Ramos-Sandoval R. Information transfer as a tool to improve the resilience of farmers against the effects of climate change: the case of the Peruvian National Agrarian Innovation System. Agric Syst. (2022) 200:103431. doi: 10.1016/j.agsy.2022.103431

[33] Aboah J Wilson MMJ Rich KM Lyne MC. Operationalising resilience in tropical agricultural value chains. Supply Chain Manag Int J. (2019) 24:271–300. doi: 10.1108/SCM-05-2018-0204

[34] Dalton TJ Yahaya I Naab J. Perceptions and performance of conservation agriculture practices in northwestern Ghana. Agric Ecosyst Environ. (2014) 187:65–71. doi: 10.1016/j.agee.2013.11.015

[35] Newsome L. Disrupted gender roles in Australian agriculture: first generation female farmers’ construction of farming identity. Agric Hum Values. (2021) 38:803–14. doi: 10.1007/s10460-021-10192-3

[36] Diallo A Dossou-Yovo ER. A gendered analysis of farmers' access to and willingness to pay for climate information services: evidence from rice farmers in Mali. Clim Serv. (2021) 35:100507. doi: 10.1016/j.cliser.2024.100507

[37] Daigle K Heiss SN. Supporting agricultural resilience: the value of women farmers’ communication practices. J Agric Food Syst Community Dev. (2020) 9:45–63. doi: 10.5304/jafscd.2020.094.010

[38] Brinkmeyer E Roesch-McNally G Dankbar H Pierre M Upton E Gwishiri N. Seeding resilience: building knowledge and capacity through relationships among black and indigenous women farmers. J Agric Food Syst Community Dev. (2025) 14:71–90. doi: 10.5304/jafscd.2025.143.025

[39] Saran A Singh S Gupta N Walke SC Rao R Simiyu C. PROTOCOL: interventions promoting resilience through climate-smart agricultural practices for women farmers: a systematic review. Campbell Syst Rev. (2022) 18:e1274. doi: 10.1002/cl2.1274, PMID: 36909889 PMC9444128

[40] Yang M Wen JX Jiao MY Xiao L. The impact of integrating agriculture and tourism on poverty reduction in China's ethnic minority areas. J Int Dev. (2025) 37:1082–103. doi: 10.1002/jid.4009

[41] Chandio AA Akram W Du AM Ahmad F Tang XP. Agricultural transformation: exploring the impact of digitalization, technological innovation and climate change on food production. Res Int Bus Finance. (2025) 75:102755. doi: 10.1016/j.ribaf.2025.102755

[42] Munthali GNC Puming H Banda LOL Ngulube PSD Daru GR Mzumara T. The effect of conservation agriculture technologies adoption on food production and security in northern Malawi: evidence from Mzimba district. Front Nutr. (2025) 12:1615990. doi: 10.3389/fnut.2025.1615990, PMID: 40704312 PMC12283979

[43] Xu JY Gu BX Tian GZ. Review of agricultural IoT technology. Art Intell Agric. (2022) 6:10–22. doi: 10.1016/j.aiia.2022.01.001

[44] Khan N Ray RL Zhang SM Osabuohien E Ihtisham M. Influence of mobile phone and internet technology on income of rural farmers: evidence from Khyber Pakhtunkhwa Province, Pakistan. Technol Soc. (2022) 68:101866. doi: 10.1016/j.techsoc.2022.101866

[45] Knickel K Ashkenazy A Chebach TC Parrot N. Agricultural modernization and sustainable agriculture: contradictions and complementarities. Int J Agric Sustain. (2017) 15:575–92. doi: 10.1080/14735903.2017.1373464

[46] Tan CF Tao JP Yi L He J Huang Q. Dynamic relationship between agricultural technology progress, agricultural insurance and farmers’ income. Agriculture. (2022) 12:1331. doi: 10.3390/agriculture12091331

[47] Wang NN Cui DF. Impact of demonstration zone policy on agricultural science and technology innovation: evidence from China. Humanit Soc Sci Commun. (2023) 10:800. doi: 10.1057/s41599-023-02292-8

[48] Wu CC Li D Zhang XQ Pan JW Quan L Yang LL. China’s agricultural machinery operation big data system. Comput Electron Agric. (2023) 205:107594. doi: 10.1016/j.compag.2022.107594

[49] Zheng YY Zhu TH Jia W. Does internet use promote the adoption of agricultural technology? Evidence from 1 449 farm households in 14 Chinese provinces. J Integr Agric. (2022) 21:282–92. doi: 10.1016/S2095-3119(21)63750-4

[50] Chen WT Hu Z-H. Using evolutionary game theory to study governments and manufacturers’ behavioral strategies under various carbon taxes and subsidies. J Clean Prod. (2018) 201:123–41. doi: 10.1016/j.jclepro.2018.08.007

[51] Luo JL Hu MJ Huang MM Bai YH. How does innovation consortium promote low-carbon agricultural technology innovation: an evolutionary game analysis. J Clean Prod. (2023) 384:135564. doi: 10.1016/j.jclepro.2022.135564

[52] Zhang F Bei JD Shi QZ Wang Y Wu L. Research on agricultural machinery services for the purpose of promoting conservation agriculture: an evolutionary game analysis involving farmers, agricultural machinery service organizations and government. Agriculture. (2024) 14:1383. doi: 10.3390/agriculture14081383

[53] Zhang SY Wang CX Yu C. The evolutionary game analysis and simulation with system dynamics of manufacturer’s emissions abatement behavior under cap-and-trade regulation. Appl Math Comput. (2019) 355:343–55. doi: 10.1016/j.amc.2019.02.080

[54] Farrokhi F Pellegrina HS. Trade, technology, and agricultural productivity. J Polit Econ. (2023) 131:2509–55. doi: 10.1086/724319

[55] Abay KA. Measurement errors in agricultural data and their implications on marginal returns to modern agricultural inputs. Agric Econ. (2020) 51:323–41. doi: 10.1111/agec.12557

[56] Barnes B Giannini F Arthur A Walker J. Optimal allocation of limited resources to biosecurity surveillance using a portfolio theory methodology. Ecol Econ. (2019) 161:153–62. doi: 10.1016/j.ecolecon.2019.03.012

[57] Afrin M Jin J Rahman A Gasparri A Tian YC Kulkarni A. Robotic edge resource allocation for agricultural cyber-physical system. IEEE Trans Netw Sci Eng. (2022) 9:3979–90. doi: 10.1109/TNSE.2021.3103602

[58] Meemken EM Aremu O Fabry A Heepen C Illien P Kammer M. Policy for decentwork in agriculture. Agric Econ. (2025) 56:401–18. doi: 10.1111/agec.70009

[59] Aubert BA Schroeder A Grimaudo J. IT as enabler of sustainable farming: an empirical analysis of farmers’ adoption decision of precision agriculture technology. Decis Support Syst. (2012) 54:510–20. doi: 10.1016/j.dss.2012.07.002

[60] Klerkx L Rose D. Dealing with the game-changing technologies of agriculture 4.0: how do we manage diversity and responsibility in food system transition pathways? Glob Food Secur. (2020) 24:100347. doi: 10.1016/j.gfs.2019.100347

[61] Tey YS Brindal M Wong SY Ardiansyah Ibragimov A Yusop MR. Evolution of precision agricultural technologies: a patent network analysis. Precis Agric. (2024) 25:376–95. doi: 10.1007/s11119-023-10076-y

[62] Engström R Nilsson M Finnveden G. Which environmental problems get policy attention?: examining energy and agricultural sector policies in Sweden. Environ Impact Assess Rev. (2008) 28:241–55. doi: 10.1016/j.eiar.2007.10.001

[63] Pelucha M Kveton V. The role of EU rural development policy in the neo-productivist agricultural paradigm. Reg Stud. (2017) 51:1860–70. doi: 10.1080/00343404.2017.1282608

[64] Severini S Di Tommaso G Finger R. Effects of the income stabilization tool on farm income level, variability and concentration in Italian agriculture. Agric Food Econ. (2019) 7:23. doi: 10.1186/s40100-019-0141-9

[65] Zhang ZY Du JT Shen ZY El Asraoui H Song ML. Effects of modern agricultural demonstration zones on cropland utilization efficiency: an empirical study based on county pilot. J Environ Manag. (2024) 349:119530. doi: 10.1016/j.jenvman.2023.119530 37948965

[66] Wu YL Wu B Liu XH Zhang SW. Digital finance and agricultural total factor productivity - from the perspective of capital deepening and factor structure. Finance Res Lett. (2025) 74:106449. doi: 10.1016/j.frl.2024.106449

[67] Meng SL Jiang YJ Song JH Sun HW Sha YF. The impact of digital inclusive finance on alternate irrigation technology innovation: from the perspective of the “catfish effect” in financial markets. Agric Water Manag. (2025) 312:109423. doi: 10.1016/j.agwat.2025.109423

[68] Yang C Liu WP Zhou JH. The role of digital finance in shaping agricultural economic resilience: evidence from machine learning. Agriculture (Basel). (2024) 14:1834. doi: 10.3390/agriculture14101834

[69] Jiang Q Li J Si H Su Y. The impact of the digital economy on agricultural green development: evidence from China. Agriculture. (2022) 12:1107. doi: 10.3390/agriculture12081107

[70] Hong X Chen Q Wang N. The impact of digital inclusive finance on the agricultural factor mismatch of agriculture-related enterprises. Financ Res Lett. (2024) 59:104774. doi: 10.1016/j.frl.2023.104774

[71] Zhang H Wang YJ Wang XQ. The impact of financial deepening on agricultural production: a household-level analysis of BigTech finance. Econ Anal Policy. (2024) 84:57–77. doi: 10.1016/j.eap.2024.08.018

[72] Miao ZY Zhou YS Ma QT. Can digital finance improve farmers’ agricultural production resilience? China Agric Econ Rev. (2025). doi: 10.1108/CAER-01-2024-0025

[73] Gao Q Sun MY Chen L. The impact of digital inclusive finance on agricultural economic resilience. Financ Res Lett. (2024) 66:105679. doi: 10.1016/j.frl.2024.105679

[74] Zhang LX Ning ZS Wang XY Luo QF Dong ZQ. Digital economy, urban-rural integration, and high-quality agricultural development. Emerg Mark Financ Trade. (2025) 61:3551–71. doi: 10.1080/1540496X.2025.2487232

[75] Lu Q Yao SR. From urban–rural division to urban–rural integration: a systematic cost explanation and Chengdu’s experience. China World Economy. (2018) 26:86–105. doi: 10.1111/cwe.12230

[76] Wang Z Liu X Qin Y Zhang Y. How rural digitization promote coordinated urban-rural development: evidence from a quasi-natural experiment in China. Agriculture. (2024) 14:2323. doi: 10.3390/agriculture14122323

[77] Liu YS Zang YZ Yang YY. China's rural revitalization and development: theory, technology and management. J Geogr Sci. (2020) 30:1923–42. doi: 10.1007/s11442-020-1819-3

[78] Odame HS Okeyo-Owuor JB Changeh JG Otieno JO. The role of technology in inclusive innovation of urban agriculture. Curr Opin Environ Sustain. (2020) 43:106–11. doi: 10.1016/j.cosust.2019.12.007

[79] Wang X Shao S Li L. Agricultural inputs, urbanization, and urban-rural income disparity: evidence from China. China Econ Rev. (2019) 55:67–84. doi: 10.1016/j.chieco.2019.03.009

[80] Shen M Ma N Chen Q. Has green finance policy promoted ecologically sustainable development under the constraints of government environmental attention? J Clean Prod. (2024) 450:141854. doi: 10.1016/j.jclepro.2024.141854

[81] Li P Lu Y Wang J. Does flattening government improve economic performance? Evidence from China. J Dev Econ. (2016) 123:18–37. doi: 10.1016/j.jdeveco.2016.07.002

[82] Fingleton B Palombi S. Spatial panel data estimation, counterfactual predictions, and local economic resilience among British towns in the Victorian era. Reg Sci Urban Econ. (2013) 43:649–60. doi: 10.1016/j.regsciurbeco.2013.04.005

[83] Bene C Frankenberger TR Nelson S Constas MA Collins G Langworthy M. Food system resilience measurement: principles, framework and caveats. Food Secur. (2023) 15:1437–58. doi: 10.1007/s12571-023-01407-y

[84] Doran J Fingleton B. US metropolitan area resilience: insights from dynamic spatial panel estimation. Environ Plan A Econ Space. (2018) 50:111–32. doi: 10.1177/0308518X17736067

[85] Bruneau M Chang SE Eguchi RT Lee GC O'Rourke TD Reinhorn AM. A framework to quantitatively assess and enhance the seismic resilience of communities. Earthquake Spectra. (2003) 19:733–52. doi: 10.1193/1.1623497

[86] Martin R Sunley P. On the notion of regional economic resilience: conceptualization and explanation. J Econ Geogr. (2015) 15:1–42. doi: 10.1093/jeg/lbu015

[87] Zheng HW Yuan ZQ Li Y Du YQ. The impact of high-standard farmland construction (HSFC) policy on green agricultural development (GAD): evidence from China. Agriculture. (2025) 15:252. doi: 10.3390/agriculture15030252

[88] Jin MM Feng Y Wang SK Chen N Cao FP. Can the development of the rural digital economy reduce agricultural carbon emissions? A spatiotemporal empirical study based on China's provinces. Sci Total Environ. (2024) 939:173437. doi: 10.1016/j.scitotenv.2024.173437, PMID: 38796024

[89] Xu JH. Spatial spillover and threshold effects of digital rural development on agricultural circular economy growth. Front Sustain Food Syst. (2024) 8:1337637. doi: 10.3389/fsufs.2024.1337637

[90] Senger I Borges JAR Machado JAD. Using the theory of planned behavior to understand the intention of small farmers in diversifying their agricultural production. J Rural Stud. (2017) 49:32–40. doi: 10.1016/j.jrurstud.2016.10.006

[91] Yuan X Zhang JL Shi J Wang JC. What can green finance do for high-quality agricultural development? Fresh insights from China. Socio Econ Plan Sci. (2024) 94:101920. doi: 10.1016/j.seps.2024.101920

[92] Okyere CY Atta-Ankomah R Asante-Addo C Kornher L. The effect of carbon farming training on food security and development resilience in northern Ghana. Clim Dev. (2025) 17:162–72. doi: 10.1080/17565529.2024.2342682

[93] Roth J Sant’Anna PHC Bilinski A Poe J. What’s trending in difference-in-differences? A synthesis of the recent econometrics literature. J Econom. (2023) 235:2218–44. doi: 10.1016/j.jeconom.2023.03.008

[94] Rambachan A Roth J. A more credible approach to parallel trends. Rev Econ Stud. (2023) 90:2555–91. doi: 10.1093/restud/rdad018

[95] Biasi B Sarsons H. Flexible wages, bargaining, and the gender gap. Q J Econ. (2022) 137:215–66. doi: 10.1093/qje/qjab026

[96] De Chaisemartin C D’Haultfoeuille X. Two-way fixed effects estimators with heterogeneous treatment effects. Am Econ Rev. (2020) 110:2964–96. doi: 10.1257/aer.20181169

[97] Goodman-Bacon A. Difference-in-differences with variation in treatment timing. J Econ. (2021) 225:254–77. doi: 10.1016/j.jeconom.2021.03.014

[98] Aghion P Akcigit U Bergeaud A Blundell R Hemous D. Innovation and top income inequality. Rev Econ Stud. (2019) 86:1–45. doi: 10.1093/restud/rdy027

[99] Boeing P Mueller E. Measuring China's patent quality: development and validation of ISR indices. China Econ Rev. (2019) 57:101331. doi: 10.1016/j.chieco.2019.101331

[100] Dechezleprêtre A Ménière Y Mohnen M. International patent families: from application strategies to statistical indicators. Scientometrics. (2017) 111:793–828. doi: 10.1007/s11192-017-2311-4, PMID: 28490823 PMC5400791

[101] Wan Q Chao RR Li JS Khan N. Toward a sustainable agricultural system in China: exploring the nexus between agricultural science and technology innovation, agricultural resilience and fiscal policies supporting agriculture. Front Sustain Food Syst. (2024) 8:1390014. doi: 10.3389/fsufs.2024.1390014

[102] Jiang BH Raza MY. Research on China’s renewable energy policies under the dual carbon goals: a political discourse analysis. Energ Strat Rev. (2023) 48:101118. doi: 10.1016/j.esr.2023.101118

[103] Dou B. Guo S. L. Chang X. C. Wang Y. Corporate digital transformation and labor structure upgrading. Int Rev Financ Anal, International Review of Financial Analysis (2023), 90,:102904. doi: 10.1016/j.irfa.2023.102904

[104] Shi Y Li ZH Lin L Chen HX Feng LJ Lu WC. From awareness to action: how climate attention drives the low-carbon transition in Chinese agriculture. J Environ Manag. (2025) 392:126700. doi: 10.1016/j.jenvman.2025.126700, PMID: 40714453

[105] Chen MH Xiao HY Zhao H Liu LA. The power of attention: government climate-risk attention and agricultural-land carbon emissions. Environ Res. (2024) 251:118661. doi: 10.1016/j.envres.2024.118661 38490628

[106] Wei SL Yang YS Ying X. Regional development, agricultural industrial upgrading and carbon emissions: what is the role of fiscal expenditure? - evidence from Northeast China. Econ Anal Policy. (2023) 80:1858–71. doi: 10.1016/j.eap.2023.11.016

[107] Allen S Badiane O Sene L Ulimwengu J. Government expenditures, health outcomes and marginal productivity of agricultural inputs: the case of Tanzania. J Agric Econ. (2014) 65:637–61. doi: 10.1111/1477-9552.12063

[108] Yang ZZ Li YL Wu CT. Population aging, fintech, and agricultural economic resilience. Int Rev Econ Finance. (2025) 97:103756. doi: 10.1016/j.iref.2024.103756

[109] Liu YS Li JT Yang YY. Strategic adjustment of land use policy under the economic transformation. Land Use Policy. (2018) 74:5–14. doi: 10.1016/j.landusepol.2017.07.005

[110] Deng WJ Hoekstra JSCM Elsinga MG. The urban-rural discrepancy of generational housing pathways: a new source of intergenerational inequality in urban China? Habitat Int. (2020) 98:102102. doi: 10.1016/j.habitatint.2019.102102

[111] Chen X Wu R. How can rural industrial revitalization and rural education level reduce the urban-rural income gap? Financ Res Lett. (2025) 73:106592. doi: 10.1016/j.frl.2024.106592

[112] Wu XG He GY. Changing ethnic stratification in contemporary China. J Contemp China. (2016) 25:938–54. doi: 10.1080/10670564.2016.1187364

[113] Wang B Li M Wen XY Yang YK Zhu JP Belzile N. Distribution characteristics, potential contribution, and management strategy of crop straw and livestock-poultry manure in multi-ethnic regions of China: a critical evaluation. J Clean Prod. (2020) 274:123174. doi: 10.1016/j.jclepro.2020.123174

