# Supplementary material for: The impact of modern agricultural technology promotion and adoption on agri-food system resilience
Source: Front Nutr. 2025 Oct 2;12:1677104. doi: 10.3389/fnut.2025.1677104 (PMC12527850; doi:10.3389/fnut.2025.1677104)
Supplement: Supplementary file 1 [file Table_1.docx]

**Appendices.**

### 1.Changing the Time Window

Robustness was tested by shortening the time window. Specifically, the study period was adjusted by excluding years prior to 2008 and years after 2020. The regression results are reported in columns (1) and (2) of Table 8. The findings show that the estimated coefficient of the interaction term (POLICY) for the NMADZ policy remains positive and significant at the 1% level. These results indicate that after the implementation of the policy, the promotion and adoption of modern agricultural technologies enhanced agri-food system resilience. This outcome is consistent with the earlier findings and confirms the robustness of the results.

### 2.Changing the Fixed Effects Structure

After controlling for year and county fixed effects in the benchmark regression, we further included city-year and province-year fixed effects in the robustness tests. The estimation results for the interaction term (POLICY) of the NMADZ policy are reported in columns (3) and (4) of Table A1. The empirical findings obtained after changing the level of fixed-effect controls are consistent with those of the benchmark regression, indicating that the promotion and adoption of modern agricultural technologies following the implementation of the policy have a stable and significant positive impact on agri-food system resilience.

### 3. Controlling for the Impact of Other Pilot Policies

During the sample period of this study, some policy interventions similar to the NMADZ policy may have confounded the regression results. In particular, the establishment of the National Agricultural Sustainable Development Pilot Demonstration Zone in 2017 and the introduction of the Agricultural Socialized Service Pilot policy in 2021 could potentially bias the findings. To control for their effects, dummy variables representing these two policies were separately added to the benchmark regression model. The corresponding results are reported in columns (5) and (6) of Table A1. After controlling for the influence of these two policies, the estimated coefficient of the interaction term (POLICY) for the NMADZ policy remains significantly positive. This indicates that the promotion and adoption of modern agricultural technologies following the implementation of the policy exert a significant positive effect on agri-food system resilience, and the benchmark regression results remain robust.

**Table A1.** Robustness Check Results.

| **Variable** | **Excluding data prior to 2008** | **Excluding data from 2020 onward** | **Re-specify the fixed effects** | **Re-specify the fixed effects** | **National Agricultural Sustainable Development Pilot Zones** | **Pilot Policy on Agricultural Socialized Services** |
| --- | --- | --- | --- | --- | --- | --- |
|  | **(1)** | **(2)** | **(3)** | **(4)** | **(5)** | **(6)** |
| POLICY | 0.1513^***^ | 0.1590^***^ | 0.1274^***^ | 0.1323^*^ | 0.1168^**^ | 0.1664^***^ |
|  | (0.0545) | (0.0529) | (0.0121) | (0.0796) | (0.0533) | (0.0401) |
| Constant | 0.6352^***^ | 0.5725^***^ | 0.6814^***^ | 0.5819^***^ | 0.5421^***^ | 0.6466^***^ |
|  | (0.0090) | (0.0075) | (0.0325) | (0.0074) | (0.0328) | (0.0418) |
| Control variable | Yes | Yes | Yes | Yes | Yes | Yes |
| Year fixed effect | Yes | Yes | No | No | Yes | Yes |
| County fixed effect | Yes | Yes | Yes | Yes | Yes | Yes |
| City-year fixed effects | No | No | Yes | No | No | No |
| Province-year fixed effects | No | No | No | Yes | No | No |
| N | 34008 | 33973 | 40565 | 40786 | 38530 | 40786 |
| R^2^ | 0.344 | 0.343 | 0.176 | 0.064 | 0.398 | 0.372 |

Standard errors in parentheses * p < 0.1, ** p < 0.05, *** p < 0.01

### 4. Quantile Regression

To eliminate potential biases arising from asymmetric distributions or multimodal patterns in the promotion and adoption of modern agricultural technologies and agri-food system resilience within the sample, this study conducts quantile regressions at the 25%, 50%, and 75% levels. The results reported in Table A2 show that the estimated coefficient of the interaction term (POLICY) for the NMADZ policy remains significantly positive. This finding indicates that after the implementation of the policy, the promotion and adoption of modern agricultural technologies significantly enhance agri-food system resilience.

### 5. Winsorization

Outliers in the data may influence the regression results and even distort the true relationships. To reduce the impact of extreme values on the results, all continuous variables were winsorized at the 1% and 99% quantiles to remove outliers and extreme values that could interfere with the regressions. The regression results, reported in Table A2, show that after eliminating outliers and extreme values, the estimated coefficient of the interaction term (POLICY) for the NMADZ policy remains significantly positive. This finding confirms the robustness of the benchmark regression results and indicates that, following the implementation of the policy, the promotion and adoption of modern agricultural technologies significantly enhance agri-food system resilience.

**Table A2.** Quantile Regression Results.

| **Variable** | **Quantile Regression** | | | **Winsorization** |
| --- | --- | --- | --- | --- |
|  | **25%** | **50%** | **75%** |  |
| POLICY | 0.1156^***^ | 0.3163^***^ | 0.0308^***^ | 0.1052^**^ |
|  | (0.0211) | (0.0396) | (0.0077) | (0.0496) |
| Constant | 0.5919^***^ | 0.8119^***^ | 0.2499^***^ | 0.5125^***^ |
|  | (0.0737) | (0.1382) | (0.0956) | (0.0833) |
| Control variable | Yes | Yes | Yes | Yes |
| Year fixed effect | Yes | Yes | Yes | Yes |
| County fixed effect | Yes | Yes | Yes | Yes |
| N | 41,616 | 41,616 | 41,616 | 41,616 |
| R^2^ | - | - | - | 0.204 |

Standard errors in parentheses * p < 0.1, ** p < 0.05, *** p < 0.01
